# Analysis of connexin 43, connexin 45 and N-cadherin in the human sertoli cell line FS1 and the human seminoma-like cell line TCam-2 in comparison with human testicular biopsies

**DOI:** 10.1186/s12885-023-10696-7

**Published:** 2023-03-10

**Authors:** Birte Schulz, Valérie Schumacher, Anaclet Ngezahayo, Daniela Maier-Begandt, Nadine Schadzek, Jochen Wilhelm, Wolfgang Weidner, Adrian Pilatz, Daniela Fietz, Sabine Kliesch, Nadine Schnepel, Nina Hambruch, Kristina Rode, Marion Langeheine, Ralph Brehm

**Affiliations:** 1 Institute of Anatomy, University of Veterinary Medicine Hannover, Foundation, Hannover, Germany; 2 Department of Urology and Medicine, Boston Children’s Hospital, Boston, MA USA; 3 Department of Surgery and Pediatrics, Harvard Medical School, Boston, MA USA; 4 Department of Cell Physiology and Biophysics, Institute of Cell Biology and Biophysics, Leibniz University Hannover, Hannover, Germany; 5 Center for Systems Neuroscience Hannover, University of Veterinary Medicine Hannover Foundation, Hannover, Germany; 6 Department of Cell Biology, Institute of Cell Biology and Biophysics, Leibniz University Hannover, Hannover, Germany; 7 Institute for Lung Health, Justus Liebig University Giessen, Giessen, Germany; 8 Universities of Giessen and Marburg Lung Center, Member of the German Center for Lung Research, Justus Liebig University Giessen, Giessen, Germany; 9 The Cardiopulmonary Institute, Justus Liebig University Giessen, Giessen, Germany; 10 Department of Urology, Pediatric Urology and Andrology, Justus Liebig University Giessen, Giessen, Germany; 11 Department of Veterinary Anatomy, Histology and Embryology, Justus Liebig University Giessen, Giessen, Germany; 12 Centre of Andrology and Reproductive Medicine, University of Muenster, Muenster, Germany

**Keywords:** FS1, TCam-2, Testicular cancer, Seminoma, Gap junctions, Connexin 43, Connexin 45, N-cadherin

## Abstract

**Background:**

Germ cell tumors are relatively common in young men. They derive from a non-invasive precursor, called germ cell neoplasia in situ, but the exact pathogenesis is still unknown. Thus, further understanding provides the basis for diagnostics, prognostics and therapy and is therefore paramount. A recently developed cell culture model consisting of human FS1 Sertoli cells and human TCam-2 seminoma-like cells offers new opportunities for research on seminoma. Since junctional proteins within the seminiferous epithelium are involved in cell organization, differentiation and proliferation, they represent interesting candidates for investigations on intercellular adhesion and communication in context with neoplastic progression.

**Methods:**

FS1 and TCam-2 cells were characterized regarding gap-junction-related connexin 43 (Cx43) and connexin 45 (Cx45), and adherens-junction-related N-cadherin using microarray, PCR, Western blot, immunocytochemistry and immunofluorescence. Results were compared to human testicular biopsies at different stages of seminoma development via immunohistochemistry to confirm the cell lines’ representativeness. Furthermore, dye-transfer measurements were performed to investigate functional cell coupling.

**Results:**

Cx43, Cx45 and N-cadherin mRNA and protein were generally detectable in both cell lines via qualitative RT-PCR and Western blot. Immunocytochemistry and immunofluorescence revealed a mainly membrane-associated expression of N-cadherin in both cell lines, but gene expression values were higher in FS1 cells. Cx43 expression was also membrane-associated in FS1 cells but barely detectable in TCam-2 cells. Accordingly, a high gene expression value of Cx43 was measured for FS1 and a low value for TCam-2 cells. Cx45 was primary located in the cytoplasm of FS1 and TCam-2 cells and revealed similar low to medium gene expression values in both cell lines. Overall, results were comparable with corresponding biopsies. Additionally, both FS1 and TCam-2 cells showed dye diffusion into neighboring cells.

**Conclusion:**

The junctional proteins Cx43, Cx45 and N-cadherin are expressed in FS1 and TCam-2 cells at mRNA and/or protein level in different amounts and localizations, and cells of both lines are functionally coupled among each other. Concerning the expression of these junctional proteins, FS1 and TCam-2 cells are largely representative for Sertoli and seminoma cells, respectively. Thus, these results provide the basis for further coculture experiments evaluating the role of junctional proteins in context with seminoma progression.

**Supplementary Information:**

The online version contains supplementary material available at 10.1186/s12885-023-10696-7.

## Introduction

Testicular tumors are the most common malignant tumors in men between 15 and 40 years and the incidence is rising [1, 2]. Germ cell tumors (GCTs) are of particular importance, of which seminoma accounts for about 50% [3]. Seminomas, as well as other human invasive GCTs, derive from a common precursor stage, called germ cell neoplasia in situ (GCNIS) [4, 5], originally described as carcinoma in situ of the testis by Skakkebaek [6]. Neoplastic initiation is thought to occur in early fetal development, presumably through an interaction of genetic and environmental factors [7, 8], but the exact cause is still unknown. GCNIS is characterized by a latent state until puberty when progression to a manifest and invasive tumor occurs [5, 9]. However, the underlying mechanisms behind this particular pathogenesis still need to be elucidated.

The seminiferous epithelium is composed of Sertoli cells (SCs) and germ cells (GCs). The tasks of SCs are manifold. Briefly, they support the development of GCs, thus enabling spermatogenesis to take place. One important task of adult SCs is the formation of the blood-testis barrier (BTB). It consists of adherens junctions (AJs), tight junctions (TJs) and gap junctions (GJs) and divides the seminiferous epithelium into a basal and an adluminal compartment [10]. GCs located in the adluminal compartment are protected from the body’s own immune system through a locally tolerogenic environment created by SCs [11].

AJs serve mechanical adhesion by connecting the membranes of two neighboring cells and establishing an association to the cytoskeleton. Different cadherins are the main group of transmembrane proteins that interact with other molecules to bind with actin filaments [12], thereby having an impact on tissue integrity and homeostasis [13]. In the human testis, neuronal (N) cadherin is the best-known cadherin [14–18]. Cadherins are further known to play an important role in differentiation and morphogenesis of tissues by being significantly involved in the migration and arrangement of cells [19]. Against this background, they also appear to participate in tumorigenesis, as the progression of a tumor is often associated with a loss of epithelial (E) cadherin and/or an increase in N-cadherin, also called cadherin switch, during the epithelial-mesenchymal transition [20, 21]. Thus, N-cadherin in particular seems to be highly involved in migration, invasion and metastasis processes [22, 23]. Along with this, expression of N-cadherin has already been shown in GCNIS and seminoma [14, 16, 17], as well as in seminoma-like TCam-2 cells [16, 23]. Therefore, N-cadherin is highly interesting for diagnostics, prognostics and therapy of testicular cancer.

GJ plaques consist of several channels which are composed of connexins [24]. These channels allow direct transfer of small molecules and ions (< 1.2 kilodalton [kDa]) between the cytoplasms of neighboring cells and thus enable direct communication [25]. Currently 21 connexins are described in humans [26], and connexin 43 (Cx43) is of special interest regarding spermatogenesis. In the human seminiferous epithelium, Cx43 is expressed both between SCs and between SCs and basal GCs [27–30]. It is first detectable with the onset of spermatogenesis during puberty, making it to a pubertal marker of differentiation [28]. The knockout of Cx43 solely in SCs leads to a loss of GCs and consequently to an arrest of spermatogenesis [31, 32], demonstrating the indispensability of Cx43 for the regular progression of spermatogenesis. Looking at it the other way round, many studies have shown that various spermatogenic disorders are associated with reduced or altered Cx43 expression [27, 28, 30, 33–35]. The situation in GCTs is similar: tubules containing GCNIS or seminoma express less or no Cx43 [29, 33, 36], so a negative correlation between the progress of tumor growth and the expression of Cx43 in the seminiferous epithelium seems to exist. Thus, the loss of intact intercellular communication and the withdrawal of negative proliferation control [37] appear to be closely linked to dedifferentiation, proliferation and invasion processes and consequently to tumor growth [29, 38]. Considering that connexins are able to suppress tumor development, they hold great hope for cancer therapy [39].

Another connexin that is thought to be present in the testis is connexin 45 (Cx45). Although it has not yet been as widely studied as Cx43, there is evidence that Cx45 occurs in the testis of different species such as mice [40–47], rats [48] and dogs [49], located in SCs, spermatocytes and round spermatids within the seminiferous epithelium [44, 48]. However, there are no information about the human testis up to now, and it is not clear to what extent Cx45 is involved in the formation of GJs in the seminiferous epithelium and thus in spermatogenesis. Furthermore, it is a still open question whether Cx45 could possibly have an influence on tumorigenesis.

The first established human SC line is the FS1 cell line [50], derived from a 19-year-old man with Frasier Syndrome in course of prophylactic orchiectomy. The cells exhibit morphological features typical for adult SCs. They further express classical SC markers and pubertal differentiation markers but not pluripotency markers or prepubertal differentiation markers, suggesting that these SCs are differentiated [50, 51]. However, a detailed investigation of cell contacts is still pending.

The TCam-2 cell line, established and characterized for the first time in 1993, is a human seminoma-like cell line [52]. Currently it is the only available seminoma-derived human cell line and is therefore of great importance for testicular cancer research. Primary cells were obtained from a 35-year-old man with a classic and pure seminoma [52]. TCam-2 cells express several markers and show morphological features that are typical for seminoma [51–57]. Only very few studies have addressed junctions until now, thus many questions are still open regarding the formation of cell contacts and the related intercellular communication.

So far, no established animal model representing GCNIS derived seminoma exists, so there is a need for alternative research approaches. Since FS1 and TCam-2 cells seem to be promising tools for coculture experiments in context with seminoma development [51, 58], we aimed to further characterize both cell lines focusing on the junctional proteins Cx43, Cx45 and N-cadherin, as intercellular adhesion and communication might play a role in progression of the precursor stage GCNIS to invasive seminoma. Up to now, the TCam-2 cell line has only been incompletely investigated for the presence of junctional proteins and the FS1 cell line not at all. To further assess the cell lines’ representativeness, comparative studies were performed with human testicular biopsies at different stages of seminoma development.

## Methods

### Cell lines and culture

The FS1 cells [50], obtained from Valérie Schumacher (Harvard Medical School, Boston, MA, USA) in September 2011, were grown in Dolbecco’s Modified Eagles’s Medium (DMEM; Invitrogen, Darmstadt, Germany) with 20% fetal calf serum (FCS), 1% penicillin/streptomycin and 2 mM l-glutamine. The TCam-2 cells [55] were kindly provided by Hubert Schorle (University of Bonn, Germany) and Daniel Nettersheim (University of Düsseldorf, Germany) in August 2011. They were grown in RPMI-1640 (Invitrogen) plus 10% FCS, 1% penicillin/streptomycin and 2 mM l-glutamine. All cells were grown at 37 °C under 5% CO_2_ and the medium was changed every two to three days. Once the cells had reached 80% confluence, they were used for further experiments. Both cell lines were tested with the PCR Mycoplasma Test Kit (AppliChem, Darmstadt, Germany) to ensure that cells are free of mycoplasma.

### Ethical statements, tissue sampling and tissue treatment

Human testicular biopsies were obtained from the Department of Urology, Pediatric Urology and Andrology at Giessen University Hospital (Wolfgang Weidner and Adrian Pilatz) and from the Center of Andrology and Reproductive Medicine at the University Medical Center of Münster (Sabine Kliesch). All subjects gave their informed consent for inclusion. The study was conducted in accordance with the Declaration of Helsinki, and the protocol was approved by the Medical Ethics Committee of the University Hospital of Giessen (decision 26/11 and 152/16). Patients were not treated with any drugs before surgery. For positive controls organs from mice were used, which were bred under a project on the SC-specific knockout of Cx43 but which genotypically corresponded to the wild type [31]. All husbandry and experimental procedures were conducted according to the German Animal Protection Law and approved by the Animal Welfare Committee of the University of Veterinary Medicine Hannover, Foundation and the Lower Saxony State Office for Consumer Protection and Food Safety (reference number 33.19-42502-05-21A575, July 2021). All experiments were performed in accordance with the relevant guidelines and regulations, including ARRIVE guidelines. Adult male mice (n = 2) were sacrificed by cervical dislocation after anesthesia with CO_2_ and the testes were surgically removed. After surgery, human and mouse specimens were fixed by immersion in Bouin’s fixative for 24 h (human tissue) or 48 h (mouse tissue) and embedded in paraffin wax with the use of standard techniques. Five-micrometer-thick sections were prepared using a microtome and stained with hematoxylin and eosin. Human testicular specimens were diagnosed by Martin Bergmann (University of Giessen, Germany) and Daniela Fietz (University of Giessen, Germany) as either histologic normal spermatogenesis (NSP), GCNIS or seminoma. Morphological and clinical features were analyzed histologically.

### Microarray analysis

Microarray analyses were performed by Jochen Wilhelm (University of Giessen, Germany) in cooperation with Cornelia Fink (University of Giessen, Germany) as described by Fink et al. (NCBI’s Gene Expression Omnibus, GEO Series accession number GSE169557) [51]. Four independent arrays were performed on samples of FS1 and TCam-2 cells after monoculture. In the present study, the focus is on the gene expression associated with junctional proteins including N-cadherin, Cx43 and Cx45, and on the expression of markers by FS1 and TCam-2 cells.

### Extraction and isolation of mRNA and protein for PCR and Western blot analysis

RNA, DNA and protein from cultured cells, which had reached approximately 80% confluence after they were grown for average three to five days, were isolated according to the TRIzol™ Reagent protocol (Invitrogen). Briefly, after removal of the medium, cultured cells were detached from the inserts with TRIzol™, transferred into a test tube and incubated at room temperature (RT). Chloroform was added, and afterwards the probes were incubated and centrifuged leading to phase separation. The aqueous phase contained the RNA, the organic lower red phenol-chloroform phase contained protein and lipids, and the interphase contained the DNA. The aqueous phase was transferred into a new test tube for RNA isolation. After adding isopropanol and incubating at RT, tubes were centrifuged. The supernatant was decanted and the remaining RNA pellet was washed with 75% ethanol and centrifuged. The new remaining pellet was separated and air-dried before resuspending in RNase-free water and incubating in a heat block at 60 °C for 15 min. The organic lower red phenol-chloroform phase and the interphase were mixed with 100% ethanol, incubated at RT and centrifuged in order to pellet the DNA. The protein-containing supernatant was removed and incubated with isopropanol at RT. After another centrifugation, the supernatant was decanted and the protein-containing pellet was washed with 0.3 M guanidine hydrochloride in 95% ethanol, incubated at RT and centrifuged. Subsequently, the pellet was resuspended in 100% ethanol, incubated at RT and centrifuged again. The now resulting protein pellet was air-dried, resuspended in 1% sodium dodecyl sulfate (SDS) and incubated at 50 °C for 1 h. Lastly, the samples were centrifuged one more time, and the supernatant containing the protein was transferred into a new tube.

Testicular biopsy specimens (NSP and GCNIS) were snap frozen in liquid nitrogen at − 80 °C. Twenty-five mg of each sample were transferred to a test tube with 500 µL TRIzol™ Reagent and homogenized using a disperser (Ultra-Turrax®, IKA, Staufen, Germany). The further steps corresponded to the TRIzol™ Reagent protocol as already described above.

### DNase digestion, cDNA synthesis (RT-PCR), PCR and sequence analysis

The concentration of the isolated RNA samples was determined via spectrometry (SmartSpec™ Plus spectrophotometer, Bio-Rad, Feldkirchen, Germany) and the sample volume was adjusted respectively. Afterwards, DNA digest was performed according to the DNase I recombinant, RNase-free (Roche, Penzberg, Germany) protocol with the optional Protector RNase Inhibitor. The RNA samples were then subjected to a reverse transcription reaction using the TaqMan® Gold RT-PCR Kit (Applied Biosystems, Foster City, CA, USA) and the MultiScribe™ Reverse Transcriptase (Invitrogen). PCR for Cx43, Cx45 and N-cadherin was performed according to the GoTaq® Flexi DNA Polymerase (Promega, Mannheim, Germany) protocol. For evaluation of RNA quality, the procedure was also performed for the reference gene beta-actin. Therefore, the same cDNA samples as for the amplification of Cx43, Cx45 and N-cadherin, respectively, were used. The primers that were applied and the annealing temperatures can be found in Table 1. Distilled water was used for negative controls instead of cDNA samples. The resulting probes were fractionated on an agarose gel and bands were visualized with GelRed® Nucleic Acid Gel Stain (Biotium, Hayward, CA, USA). To record the images, the UV transilluminator Bio-Vision-3026 WL/26 M was used in combination with the software VisionCapt 15.06 (Vilber Lourmat, Eberhardzell, Germany). Sequencing of PCR products was done by Seqlab Sequencing Service (Seqlab, Göttingen, Germany).

**Table 1:** Primers used for PCR analysis and according annealing temperatures

Target gene	Primer	Primer sequence (5’→3’)	Length	Accession no./ reference	Annealing temperature
*CDH2* (N-cadherin)	Forward Reverse	TCCACCATATGACTCCCTGTTAGT CAGAAAACTAATTCCAATCTGAAA	454 bp	NM_001792.5 [59]	55 °C
*GJA1* (Connexin 43)	Forward Reverse	CCATCTCTAACTCCCATGCACAGC TGGCACGACTGCTGGCTCTGCTT	138 bp	NM_000165.5 [60]	60 °C
*GJC1* (Connexin 45)	Forward Reverse	ATGAGTTGGAGCTTCCTGACTCG CGGCTGTTCTGTGTTGCAC	174 bp	NM_005497.4 [61]	65 °C
*ACTB* (Beta-actin)	Forward Reverse	TTCCTTCCTGGGCATGGAGT TACAGGTCTTTGCGGATATC	90 bp	NM_001101.5	60 °C

### Qualitative Western blot analysis

The protein concentration of each cell line sample was determined via the DC™ Protein Assay Kit II (Bio-Rad) and its respective protocol using Mithras Multimode Microplate Reader LB 940 (Berthold Technologies, Bad Wildbad, Germany) and a 700 nm absorbance filter. All protein samples were adjusted to 20 µg using a 4 x SDS and Laemmli buffer and incubated at 40 °C for 5 min. The proteins were then separated via SDS polyacrylamide gel electrophoresis (PAGE): a stacking gel and a 10% resolving gel. The SDS-PAGE gel was run using the PerfectBlue™ dual gel system Twin S (Peqlab Biotechnologies, Erlangen, Germany) at 100 volts for 10 min and then additional 90–100 min at 150 volts in a SDS running buffer. Neatly, proteins were blotted onto a Protran BA 85 nitrocellulose membrane (Whatman, Dassel, Germany) using the PerfectBlue™ Electroblotting Systems Sedec M (Peqlab) at 1 mA/cm^2^ for 1 h in blotting buffer. The membrane was then blocked with 5% non-fat dry milk in tris-buffered saline with Tween (TBS-T) for 1 h on a shaker. All following steps were performed on a shaker as well. To remove remaining non-fat dry milk, the membrane was washed in TBS-T and after this incubated at 4 °C overnight with the respective primary antibody against Cx43, Cx45 or N-cadherin (Table 2) diluted in TBS. Negative controls were processed without the primary antibody and incubated only with TBS instead. Since the entire membrane was coated with the primary antibody, negative controls were performed on separate membranes but were otherwise treated in the same way simultaneously. On the next day, after washing the membrane in TBS, the appropriate secondary antibody was added (Table 2) and incubated at RT for 45 min. Afterwards, the membrane was washed again. To visualize the chemiluminescence, the SuperSignal™ West Dura Extended Duration Substrate (Thermo Fisher Scientific, Bonn, Germany) was used. The chemiluminescence was recorded with the use of a Fusion-SL 3500-WL and the softwares Fusion 15.09 and Bio-1D 12.11 (Vilber Lourmat). To detect the loading control beta-actin, which was used to ensure that the protein loads were distributed evenly, the blotted membrane was washed in TBS, incubated in stripping buffer containing SDS, glycin and Tween-20 and then washed in TBS-T and in TBS. All other processing steps for beta-actin were performed as described previously.

**Table 2:** Antibodies used for Western blot (WB), immunocytochemistry (ICC) or immunohistochemistry (IHC) and immunofluorescence (IF).

	Protein	Material	Primary antibody	Dilution	Secondary antibody
**WB**	**N-cadherin**	FS1, TCam-2	Mouse monoclonal antibody, 33-3900, Invitrogen	1:500	Goat anti-mouse IgG-HRP, sc-2005, Santa Cruz, 1:5000
	**Connexin 43**	FS1, TCam-2	Rabbit polyclonal antibody, 3512, NEB * / Cell Signaling	1:1000	Goat anti-rabbit IgG-HRP, sc-2004, Santa Cruz, 1:5000
	**Connexin 45**	FS1, TCam-2	Rabbit polyclonal antibody, T. Steinberg, St. Louis [62–64]	1:1000	Goat anti-rabbit IgG-HRP, sc-2004, Santa Cruz, 1:5000
	**Beta-actin**	FS1, TCam-2	Mouse monoclonal antibody, sc-47,778, Santa Cruz	1:5000	Goat anti-mouse IgG-HRP, sc-2005, Santa Cruz, 1:5000
**ICC/ IHC**	**N-cadherin**	FS1, TCam-2	Mouse monoclonal antibody, 33-3900, Invitrogen	1:1000 ^†^ 1:2000 ^‡^	HRP Labelled Polymer Anti-Mouse, K4001, Dako, ready to use
		Testis	Rabbit polyclonal antibody, ab76057, Abcam	1:1500 ^§^ 1:3000 $^\parallel$	HRP Labelled Polymer Anti-Rabbit, K4003, Dako, ready to use
	**Connexin 43**	FS1, TCam-2	Rabbit polyclonal antibody, 3512, NEB * / Cell Signaling	1:100	HRP Labelled Polymer Anti-Rabbit, K4003, Dako, ready to use
		Testis	Rabbit polyclonal antibody, 3512, NEB * / Cell Signaling	1:500	HRP Labelled Polymer Anti-Rabbit, K4003, Dako, ready to use
	**Connexin 45**	FS1, TCam-2	Rabbit polyclonal antibody, T. Steinberg, St. Louis [62–64]	1:6000	HRP Labelled Polymer Anti-Rabbit, K4003, Dako, ready to use
		Testis	Rabbit polyclonal antibody, CX45B12-A, Alpha Diagnostic	1:1000	HRP Labelled Polymer Anti-Rabbit, K4003, Dako, ready to use
**IF**	**N-cadherin**	FS1, TCam-2	Mouse monoclonal antibody, 33-3900, Invitrogen	1:1000	Alexa Fluor 488 anti-Mouse, A-11,001, Invitrogen, 1:5000
	**Connexin 43**	FS1, TCam-2	Rabbit polyclonal antibody, 3512, NEB * / Cell Signaling	1:100	Alexa Fluor 488 anti-Rabbit, A-11,008, Invitrogen, 1:5000
	**Connexin 45**	FS1, TCam-2	Rabbit polyclonal antibody T. Steinberg, St. Louis [62–64]	1:6000	Alexa Fluor 488 anti-Rabbit, A-11,008, Invitrogen, 1:5000

* New England Biolabs; ^†^ dilution for FS1; ^‡^ dilution for TCam-2; ^§^ dilution for human samples representing normal spermatogenesis and germ cell neoplasia in situ; $^\parallel$ dilution for seminoma.

### Immunocytochemistry and immunofluorescence

Cells were seeded out on glass cover slides at 15,000 cells per well and incubated at 37 °C and 5% CO_2_ for one week. After removal of the medium and washing with TBS, cells were fixated with methanol at RT for 10 min. Afterwards, the cover slides were changed over in glass dishes and fixed with baysilone paste (Bayer, Leverkusen, Germany). The fixated cells were then washed with TBS-T and blocked at RT for 45 min using 5% non-fat dry milk in TBS-T. The solution was removed and the appropriate primary antibody against Cx43, Cx45 or N-cadherin (Table 2) diluted in TBS was incubated at 4 °C overnight. Negative controls were treated with TBS-T only.

For immunocytochemistry, the next day, cells were washed in TBS. Visualization was performed according to a slightly modified protocol of the EnVision™+ System- HRP (Dako, Hamburg, Germany) in combination with the Liquid DAB + Substrate Chromogen System (Dako). Briefly, the appropriate secondary antibody (Table 2) was added for 20 min. After the antibody had been washed off with TBS, DAB + Substrate Buffer and DAB + Chromogen solution (mixed according to protocol) were added and washed off again as soon as the reaction became visible. Afterwards, cells were counterstained with hemalum to identify the nuclei and washed with tap water. The glass cover slides were then fixated with gelatin onto a microscope slide. Cells were viewed and photographed with a Zeiss Axioskop microscope (Carl Zeiss, Oberkochen, Germany) and a SC 50 Camera (Olympus, Hamburg, Germany) using the cellSens Standard 2.2 software (Olympus).

For immunofluorescence respectively, an Alexa Fluor® secondary antibody (Table 2) diluted in TBS was added for 45 min at 4 °C in the dark (all following steps were performed in darkness as well). After the antibody had been washed off, the nuclei were stained with Hoechst 33,342 (Invitrogen) diluted 1:8000. After further washings in TBS and sterile water, glass cover slides were fixated with ProLong™ Gold Antifade Reagent (Invitrogen) onto a microscope slide. Cells were viewed and photographed with a Zeiss Axiovert 200 M fluorescence microscope and a Zeiss AxioCam MR3 camera using the software Axio Vision 4.8 (Carl Zeiss).

### Dye-transfer measurements

Analysis of functional GJ coupling was performed as previously described [65]. Briefly, TCam-2 and FS1 cells were grown on cover slips for two to three days. A cover slip was introduced into a perfusion chamber mounted on an inverted fluorescence microscope (Carl Zeiss) and containing 500 µL of a bath solution composed of (in mM) 140 NaCl, 5 KCl, 10 HEPES, 1 MgCl_2_, 10 glucose and 2 CaCl_2_ (pH 7.4). A whole-cell patch-clamp configuration was established on one cell (donor cell) in close vicinity to other cells (acceptor cells) using an EPC7 patch-clamp amplifier (List Medical, Darmstadt, Germany). Lucifer Yellow lithium salt (LY; 1 mg/mL; Biotium) was added to the pipette medium composed of (in mM) 135 K-gluconate, 5 KCl, 10 HEPES, 1 MgCl_2_, 2 glucose, 5 Na_2_ATP, 5 EGTA, 0.1 cAMP and 0.1 cGMP (pH 7.4). To track the loading of LY into the donor cell, and to determine the potential dye diffusion trough GJs into the acceptor cells, the fluorescence of LY was recorded at 540–580 nm with a digital CCD camera (C4742-95, Hamamatsu Photonics K.K., Hamamatsu, Japan) after excitation at 410 nm with a Polychrome II monochromator (T.I.L.L. Photonics GmbH, Planegg, Germany) equipped with a 75 W XBO xenon arc lamp. For the control of the monochromator and of the recording camera the software Aquacosmos (Hamamatsu Photonics K.K.) was used. The cells were considered as functionally coupled if LY fluorescence was observed in at least one of the acceptor cells within 10 min following the establishment of the whole-cell patch-clamp configuration on the donor cell.

### Immunohistochemistry

Human or mouse tissue sections were first deparaffinized using a descending alcohol series. Thereby the sections were treated in 196 mL of 80% alcohol together with 4 mL of 30% hydrogen peroxide for 30 min. After washing in TBS-T, the samples were pre-treated with citrate buffer at 96–99 °C for 20 min. After further washings, 3% bovine serum albumin (BSA) in phosphate-buffered saline (PBS) was added. Then, the appropriate primary antibody for Cx43, Cx45 or N-cadherin (Table 2) diluted in 1% BSA/PBS was incubated overnight at 4 °C. Negative controls were treated with only 1% BSA/PBS instead of primary antibodies. As part of the experiments targeting Cx45, a follow-up section from each tissue was additionally treated with control peptide (CX45B12-P, Alpha Diagnostic, San Antonio, TX, USA) which was diluted 1:100 in the primary antibody solution. The next day, the primary antibody was first washed off with PBS, and then the appropriate secondary antibody (Table 2) was applied for 30 min. After the antibody had been removed with PBS, DAB + Substrate Buffer and DAB + Chromogen solution were added and washed off with PBS again as soon as the reaction became visible. After counterstaining with hemalum and washing under running tap water, the sections were treated with an ascending alcohol series and finally covered with Eukitt® (ORSAtec, Bobingen, Germany). The tissue was viewed and photographed with a Zeiss Axioskop microscope (Carl Zeiss) and a SC 50 Camera (Olympus) using the cellSens Standard 2.2 software (Olympus).

## Results

### N-cadherin, Cx43 and Cx45 are expressed at mRNA level in FS1 and TCam-2 cells

**Fig. 1:**
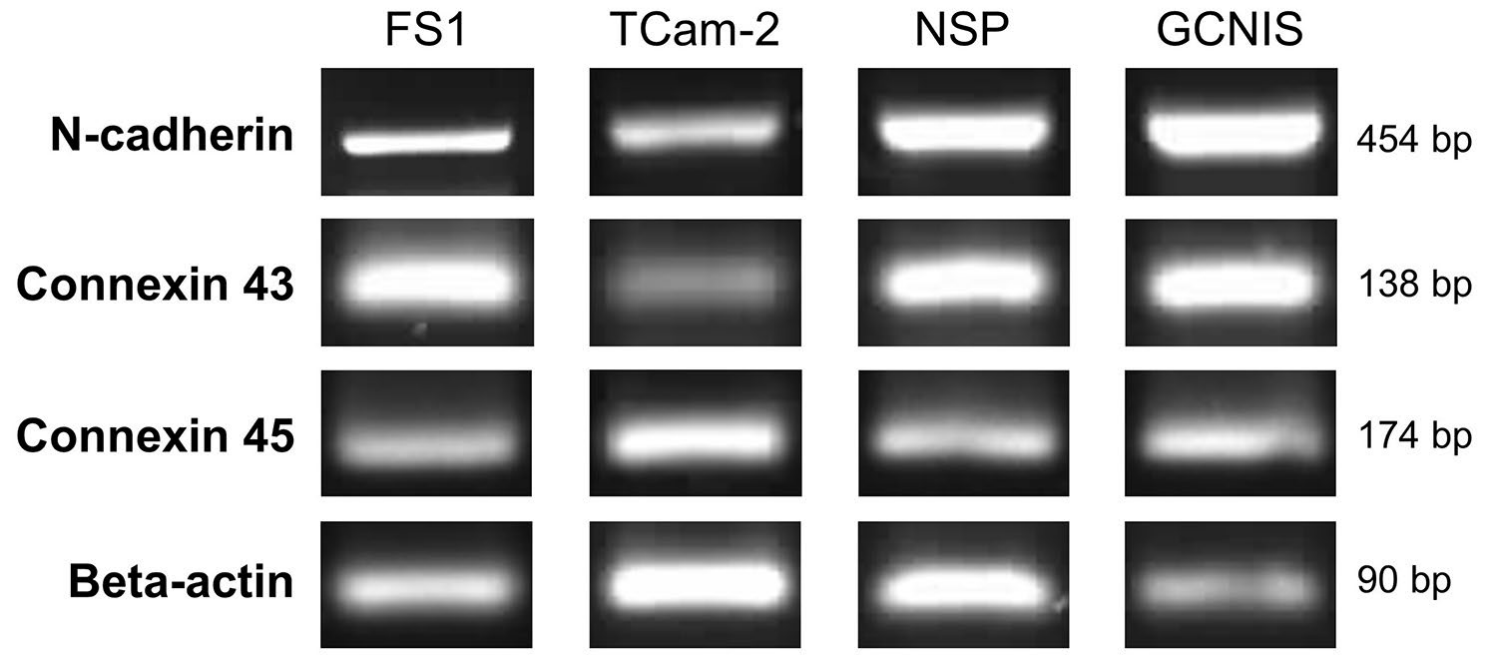
Qualitative PCR analysis of selected junctional proteins in FS1 and TCam-2 cells and human testicular specimens. This figure is only intended to provide an overview of the qualitative occurrence of the different mRNAs in FS1 and TCam-2 cells and not to provide a quantitative comparison. The cropped bands, which are shown in this figure, clearly separated from each other by spaces, originate from different experiments for each protein and cell line, which were carried out under the same conditions in a narrow timeframe. The size of each band in base pairs (bp) is indicated on the right side. Bands corresponding to N-cadherin (454 bp), connexin 43 (138 bp), connexin 45 (174 bp) and beta-actin (90 bp) are detectable in FS1 and TCam-2 cells, as well as in human testicular samples representing normal spermatogenesis (NSP) and germ cell neoplasia in situ (GCNIS).

N-cadherin, Cx43 and Cx45 could be shown at mRNA level for FS1, TCam-2, NSP and GCNIS samples by PCR analysis (Fig. 1). The typical band for N-cadherin at 454 base pairs [bp] appeared both in samples of FS1 and TCam-2 cells, as well as in homogenized testicular biopsy specimens from patients with NSP and GCNIS, respectively. For Cx43 likewise a 138 bp PCR product was observed for FS1, TCam-2, NSP and GCNIS samples. A PCR product for Cx45 at 174 bp was clearly visible in all samples investigated. In addition, the band for beta-actin, a reference gene, could be identified in every sample as well, represented by a single band at approximately 90 bp. As cropped images are presented in Fig. 1, full-length gels are deposited in Additional file 1 (Supplementary Figs. 1–8).

**Fig. 2:**
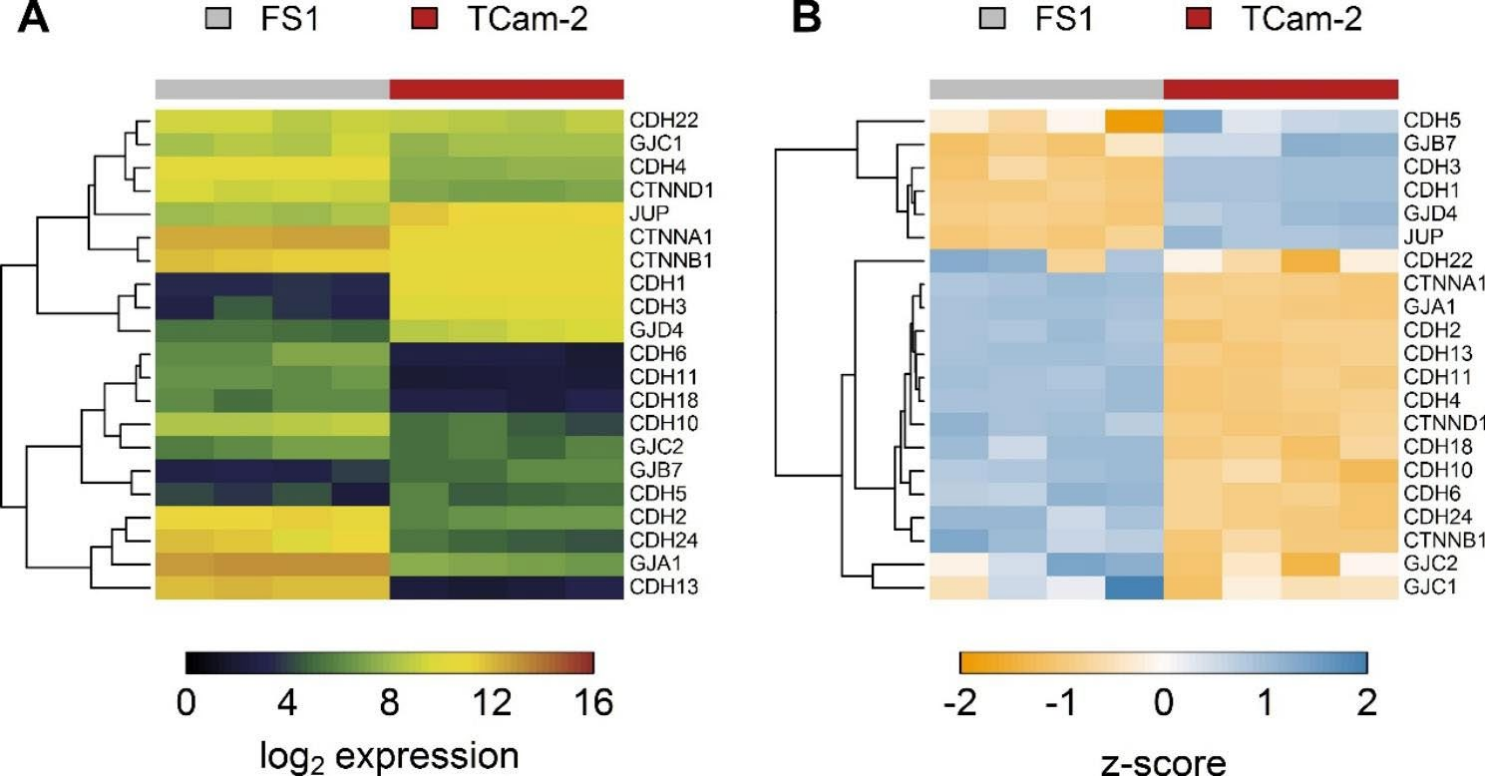
Expression of genes related with adherens and gap junctions in FS1 and TCam-2 cells. The heatmaps show results for the microarray analysis of monocultured FS1 and TCam-2 cells. Columns represent biological replicates and rows a selection of expressed genes. Only genes that are expressed in at least one of the two cell lines are listed. Heatmap (**A**) shows expression values and heatmap (**B**) shows z-scores

Microarray analysis revealed an average gene expression value (arithmetic mean of the average log_2_ spot signal intensity of four independent arrays for each cell line rounded to three decimal places) of 11.161 for N-cadherin (*CDH2*) and 13.05 for Cx43 (*GJA1*) in FS1 cells, what means a high expression of both genes. In contrast, the expression of Cx45 (*GJC1*) was only 8.553, reflecting a medium expression. For TCam-2 cells, an expression value of 6.362 for N-cadherin (*CDH2*), 7.03 for Cx43 (*GJA1*) and 8.007 for Cx45 (*GJC1*) was measured, representing low to medium expression levels. These results are visualized in Fig. 2, whereby heatmap A (Fig. 2A) represents expression values and heatmap B (Fig. 2B) z-scores; other genes associated with AJs and GJs that are expressed in at least one of the two cell lines are listed as well. Additionally, Supplementary Fig. 9 (Additional file 2) shows the expression values of certain known markers within FS1 and TCam-2 cells.

### N-cadherin, Cx43 and Cx45 protein is detectable in FS1 and TCam-2 cells

**Fig. 3:**
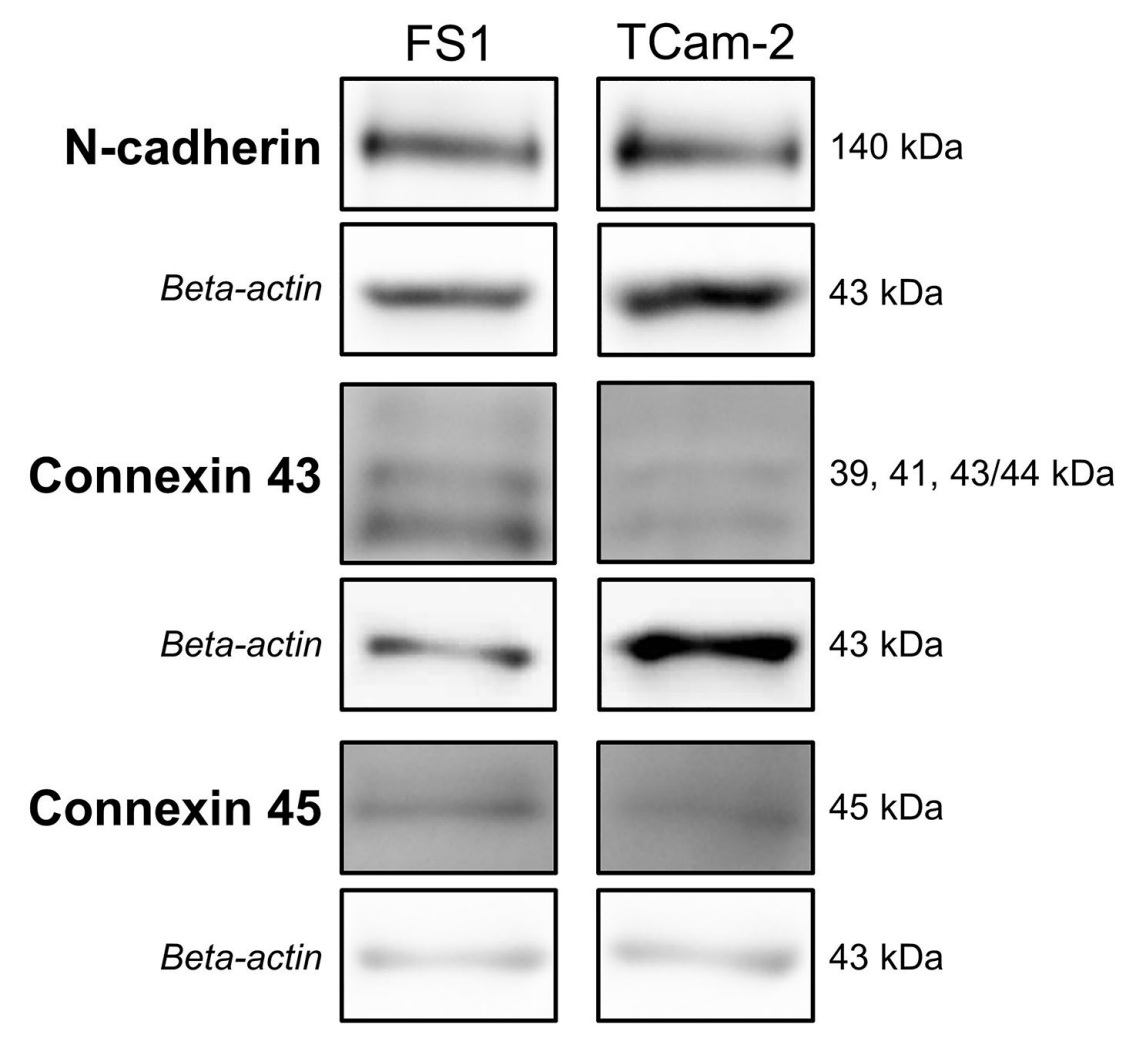
Qualitative Western blot analysis of selected junctional proteins in FS1 and TCam-2 cells. The corresponding loading control (beta-actin at 43 kDa) is shown below respectively, which was run each on the same membrane after treatment with stripping buffer. This figure is only intended to provide an overview of the qualitative occurrence of the different proteins in FS1 and TCam-2 cells and not to provide a quantitative comparison. The cropped bands, which are shown in this figure, clearly separated from each other by black frames and spaces, originate largely from different experiments, which were carried out under the same conditions in a narrow timeframe, and the exposure times vary slightly. The molecular weight of each band is indicated on the right side. Bands corresponding to N-cadherin (140 kDa), connexin 43 (39–44 kDa) and connexin 45 (45 kDa) are detectable in FS1 and TCam-2 cells

N-cadherin, Cx43 and Cx45 could be shown at protein level for FS1 and TCam-2 cells via Western blot analysis (Fig. 3). For N-cadherin both FS1 and TCam-2 samples revealed a clear immunoreactive band at 140 kDa. On the contrary, three characteristic bands at 39, 41 and 43/44 kDa—probably representing different phosphorylated isoforms of Cx43—could be demonstrated for FS1 cells. In case of TCam-2, only two bands at 39 and 41 kDa were detectable. The immunoreactive band at 45 kDa corresponding to Cx45 also occurred for both cell lines. Beta-actin, a protein that served as a loading control, could always be observed in all samples investigated, based on an immunoreactive band at 43 kDa. As cropped images are presented in Fig. 3, full-length blots are deposited in Additional file 3 (Supplementary Figs. 10–15).

### Different expression patterns of N-cadherin, Cx43 and Cx45 in FS1 and TCam-2 cells

**Fig. 4:**
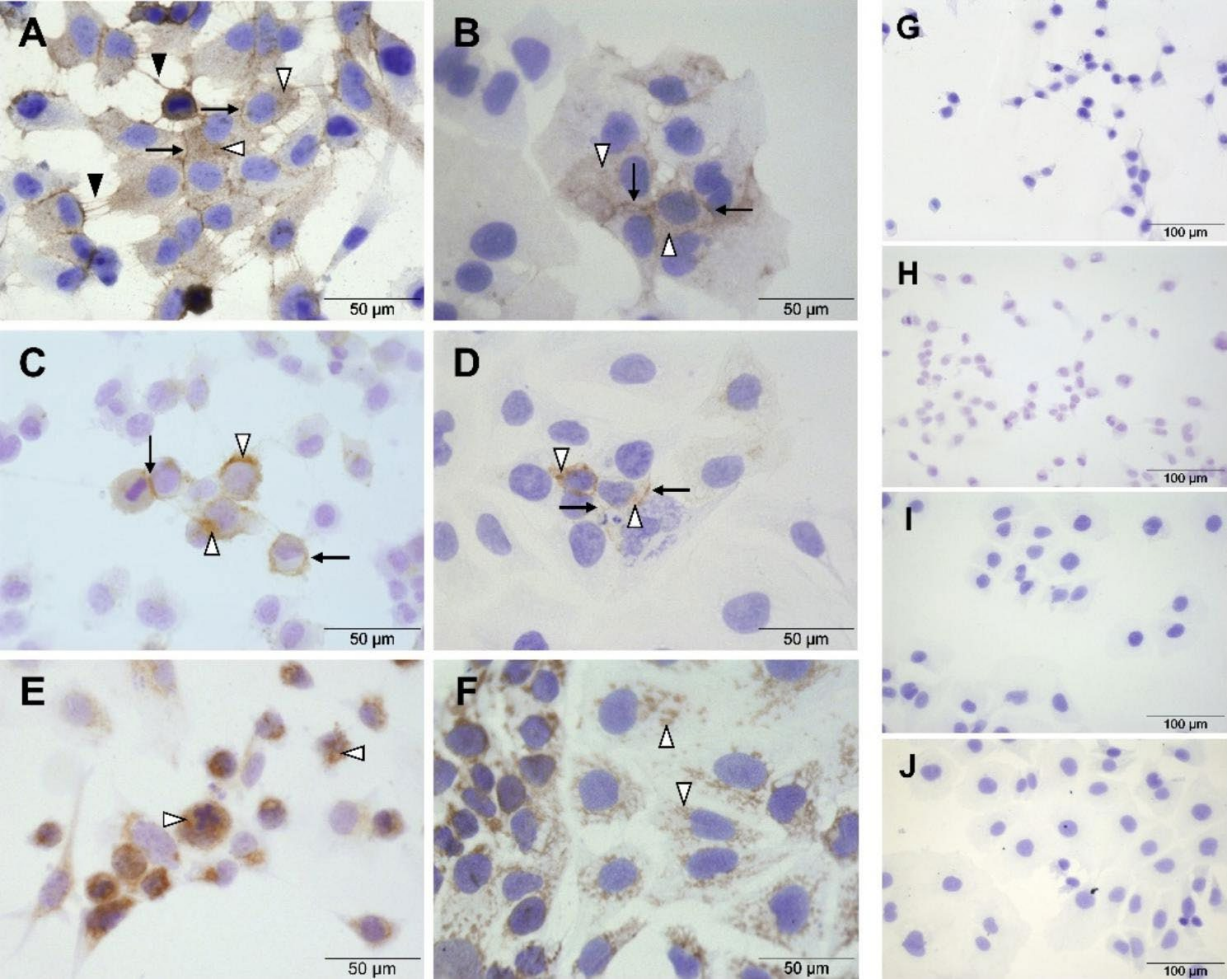
Immunostaining of N-cadherin, connexin 43 and connexin 45 in cultured FS1 and TCam-2 cells. (**A**) In FS1 cells, the N-cadherin representing signal is particularly localized within the cell membranes (arrows) and cell extensions (black arrowheads), but it can also be detected in the cytoplasm at a few sites (white arrowheads). (**B**) Similarly, in TCam-2 cells, N-cadherin can be detected at the cell membranes (arrows) and to a lesser extent in the cytoplasm (white arrowheads). (**C**) The labelling of connexin 43 in FS1 cells is mainly membrane-bound (arrows), but a very weak signal in the cytoplasm can be observed at only a few areas (white arrowheads). (**D**) TCam-2 cells show just a very weak reaction for connexin 43 overall, which can only be found in some cells with both membrane (arrows) and cytoplasm (white arrowheads) being positive. (**E**, **F**) Both within FS1 cells (**E**) and TCam-2 cells (**F**), the signal of the immune reaction for connexin 45 is present in the cytoplasm in a dot-like pattern (white arrowheads). The negative controls, treated without any primary antibody but with the secondary antibodies anti-mouse (**G**, **I**) or anti-rabbit (**H**, **J**), show no brown labelling for either FS1 cells (**G**, **H**) or TCam-2 cells (**I**, **J**). Scale bars = 50 μm (**A**–**F**), 100 μm (**G**–**J**).

**Fig. 5:**
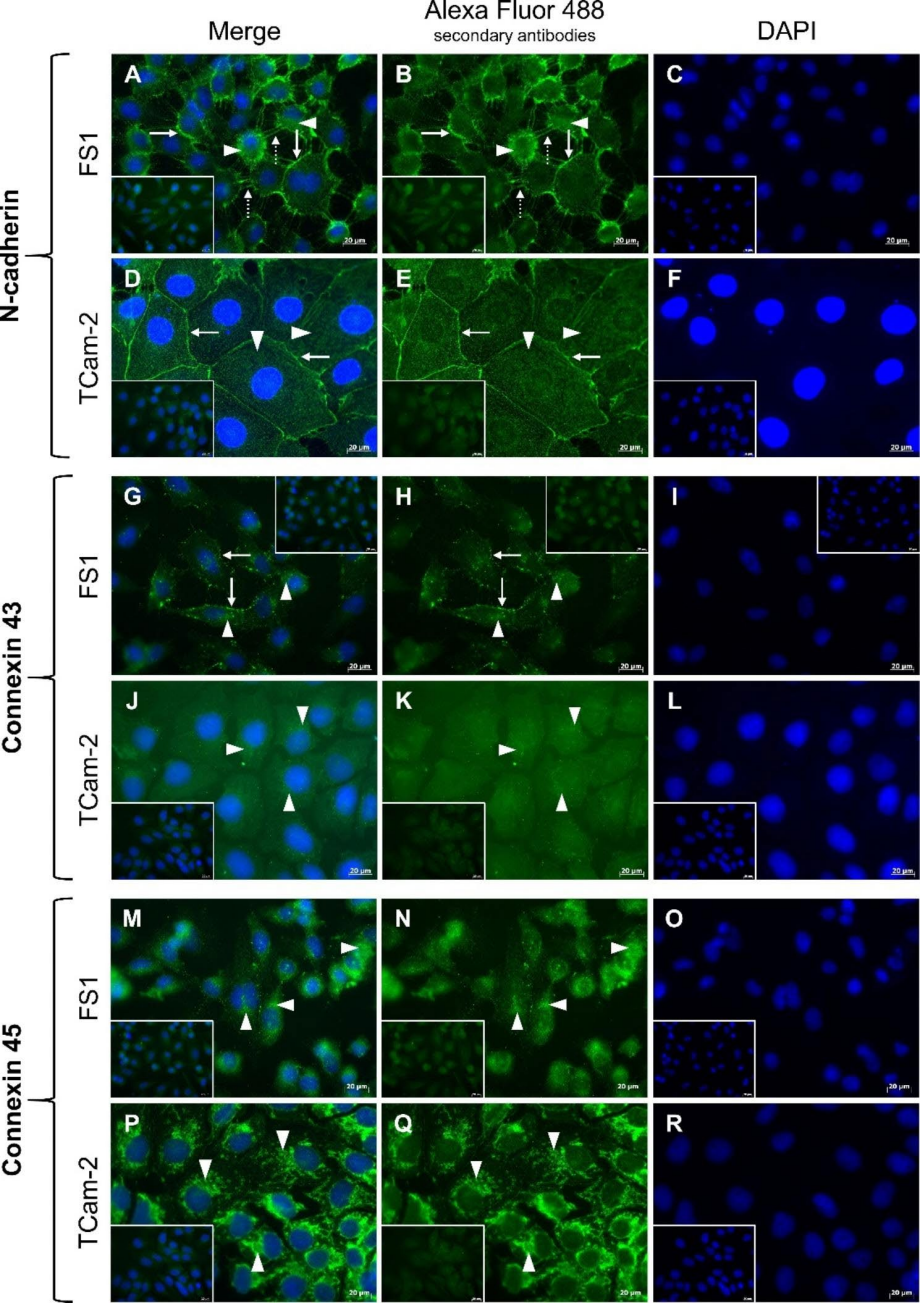
Immunofluorescence labelling of selected junctional proteins in cultured FS1 and TCam-2 cells. (**A**–**C**) The immunosignal corresponding to N-cadherin is visible at the cell membranes (arrows) and cell extensions (dashed arrows) of FS1 cells and to a lesser extent in the cytoplasm (arrowheads). (**D**–**F**) A similar pattern occurs in TCam-2 cells with mainly the membranes (arrows) but also the cytoplasm (arrowheads) fluorescing. (**G**–**I**) A punctate fluorescent signal representing connexin 43 is localized at the cell membranes of FS1 cells (arrows), but areas of the cytoplasm are positive as well (arrowheads). (**J**–**L**) TCam-2 cells present only a weak reaction in the cytoplasm, especially perinuclear (arrowheads). (**M**–**O**) The fluorescent signal for connexin 45 is localized diffusely in the cytoplasm of FS1 cells, producing a dot-like pattern (arrowheads). (**P**–**R**) Similarly, in TCam-2 cells, the signal is distributed irregularly in the cytoplasm (arrowheads). The negative controls (**inserts**) do not show any specific labelling; only a weak fluorescence of the background can be observed. Scale bars = 20 μm

The immunocytochemical analysis of N-cadherin in cultured FS1 cells resulted in a clearly visible immunoreaction at the cell membranes (Fig. 4A, arrows) and cell extensions (Fig. 4A, black arrowheads). In addition, an immunopositive signal was detectable in the cytoplasm at a few sites (Fig. 4A, white arrowheads). The TCam-2 cells also displayed a stronger staining at the cell membranes (Fig. 4B, arrows) and a weaker staining diffusely distributed in the cytoplasm of some cells (Fig. 4B, white arrowheads). These results were confirmed by immunofluorescence. Again, N-cadherin revealed a strong membrane labelling in both FS1 cells (Fig. 5A–C, arrows) and TCam-2 cells (Fig. 5D–F, arrows). FS1 cells also showed a signal localized at the cell extensions (Fig. 5A–C, dashed arrows). In addition, in cells of both cell lines, the cytoplasm was diffusely positive for the reaction (FS1: Fig. 5A–C, arrowheads; TCam-2: Fig. 5D–F, arrowheads) but with a weaker intensity compared to the signal localized at the membranes.

In comparison, immunocytochemical detection of Cx43 in FS1 cells resulted in a membrane-bound staining (Fig. 4C, arrows) and a very weak signal in the cytoplasm at only a few sites (Fig. 4C, white arrowheads). Immunofluorescence further revealed that the immunopositive signal was distributed in a punctate manner along the cell membranes (Fig. 5G–I, arrows) and confirmed the low-level cytoplasmic reactivity within FS1 cells (Fig. 5G–I, arrowheads). Within TCam-2 cells, the immunocytochemical reaction for Cx43 was very weak and only visible in some cells. It appeared to occur both at the membrane (Fig. 4D, arrows) and in the cytoplasm (Fig. 4D, white arrowheads). In contrast, the immunofluorescence reaction was limited to the cytoplasm of TCam-2 cells, and the signal appeared to be slightly stronger in the perinuclear region (Fig. 5J–L, arrowheads).

Cx45 could be detected by immunocytochemistry both within FS1 (Fig. 4E, white arrowheads) and within TCam-2 cells (Fig. 4F, white arrowheads) but only in the cytoplasm. The staining pattern appeared to be granular to dot-like and was distributed diffusely. Using immunofluorescence, a similar cytoplasmic reaction pattern was achieved within FS1 (Fig. 5M–O, arrowheads) and TCam-2 cells (Fig. 5P–R, arrowheads).

Regarding immunocytochemistry, the negative controls, treated without any primary antibody but with the secondary antibodies anti-mouse (Fig. 4G, I) or anti-rabbit (Fig. 4H, J), showed no immunopositive labelling for either FS1 cells (Fig. 4G, H) or TCam-2 cells (Fig. 4I, J). Equally, in case of immunofluorescence, the negative controls did not show any specific labelling; only a weak fluorescence of the background could be observed (Fig. 5, inserts).

### Both FS1 and TCam-2 cells are functionally coupled among each other

**Fig. 6:**
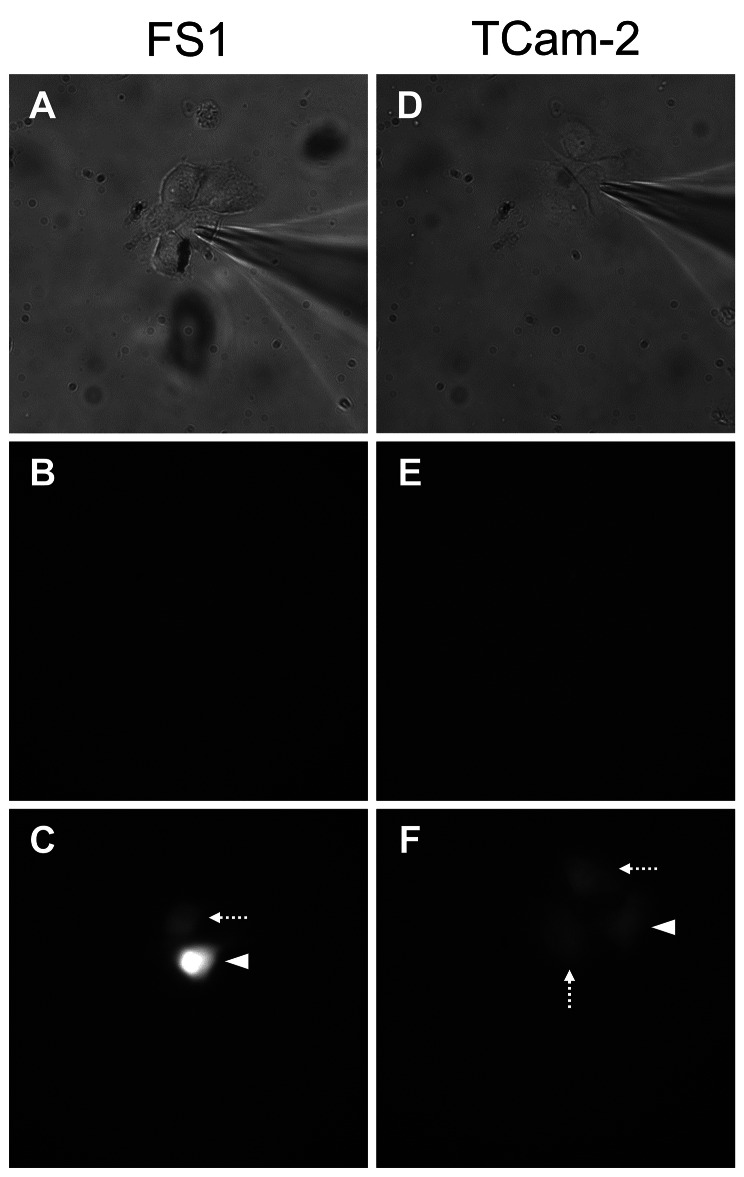
Dye coupling experiments. A whole-cell patch-clamp configuration was established with a Lucifer Yellow (LY) containing pipette-filling solution (1 mg/mL) into one FS1 cell (**A**–**C**) and one TCam-2 cell (**D**–**F**). The images are representative images. Phase contrast images of the respective cell line before microinjection (**A**, **D**) and fluorescence images prior (**B**, **E**) and after LY-microinjection and incubation (**C**, **F**) are illustrated. Cell coupling of both cell lines FS1 (**C**) and TCam-2 (**F**) can be seen in acceptor cells (dashed arrows) neighboring the donor cells (arrowheads), visualized by the fluorescent LY.

After microinjection of the dye LY into the respective donor cell (Fig. 6A and D) and subsequent incubation for 10 min, the diffusion of the dye was assessed by its fluorescence (Fig. 6C and F). Before incubation (Fig. 6B and E) no fluorescent signal could be observed. After incubation, if diffusion into one or more neighboring cells was successful, the fluorescent dye became visible not only in the donor cell (Fig. 6C and F, arrowheads) but also in the corresponding recipient cell(s) (Fig. 6C and F, dashed arrows). When investigating the FS1 cell line, 11 out of 14 tested cells (79%) showed a connection to at least one neighboring cell, for TCam-2 cells, nine out of 16 tested cells (56%) did (Table 3).

**Table 3:** Number of cells tested and coupled via gap junctions after dye coupling experiments.

Cell line	Number of cells tested	Number of cells coupled
FS1	14	11
TCam-2	16	9

### Comparable expression patterns of N-cadherin, Cx43 and Cx45 in corresponding human testicular biopsies

**Fig. 7:**
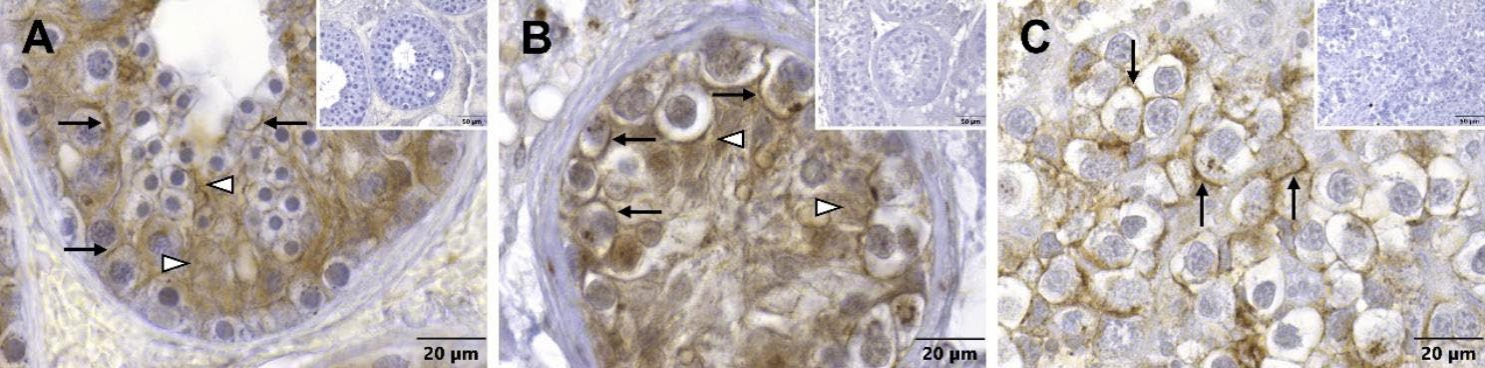
Immunostaining of N-cadherin in human testicular biopsy specimens. (**A**) In the seminiferous tubule with normal spermatogenesis, the brown signal of the reaction can be mainly seen in the cytoplasm of Sertoli cells (white arrowheads), which surround different germ cells, but also the cell boundaries between adjacent Sertoli and germ cells are in parts clearly stained (arrows). (**B**) Tubules harboring germ cell neoplasia in situ also show distinct staining of the cytoplasm of Sertoli cells (white arrowheads), which are displaced to the center of the tubule. The basally located degenerated germ cells display staining of the cell membranes (arrows). (**C**) The membranes of adjacent seminoma cells show a positive signal (arrows), while Sertoli cells cannot be found anymore. Negative controls (**inserts**) do not show any reaction. Scale bars = 20 μm (**A**–**C**), 50 μm (**inserts**)

In seminiferous tubules with NSP, N-cadherin was clearly detectable in the cytoplasm of SCs (Fig. 7A, white arrowheads), which surround different GCs and extend from the basal to the adluminal compartment. In some places, it appeared that the immunopositive signal was concentrated particularly at the boundaries between SCs and GCs (Fig. 7A, arrows), but it is difficult to tell whether it was associated with the membranes of SCs or GCs. A similar staining pattern was observed in seminiferous tubules from patients with GCNIS. Here too the reaction seemed to have taken place mainly in the cytoplasm of SCs, which had been displaced to the center of the tubule (Fig. 7B, white arrowheads). Staining also appeared to be concentrated at the borders between SCs and basally located degenerated GCs (Fig. 7B, arrows). It should be kept in mind that the cytoplasm of the carcinoma cells is lost during treatment of paraffin sections due to the high glycogen content, so that no statement can be made about the presence of the examined proteins in the cytoplasm. In case of seminoma, which no longer showed any tubules or SCs, a clear staining of the membranes of adjacent tumor cells created a comb-like pattern (Fig. 7C, arrows). The respective negative controls did not display any staining (Fig. 7, inserts).

**Fig. 8:**
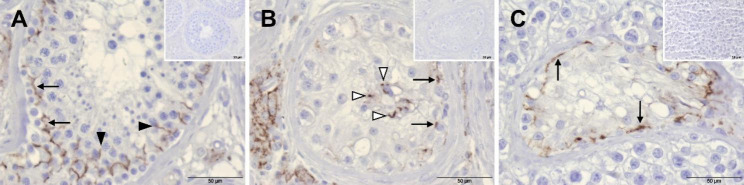
Immunostaining of connexin 43 in human testicular biopsy specimens. (**A**) In the seminiferous tubule with normal spermatogenesis, a linear staining pattern can be observed in basal regions above spermatogonia (arrows), which partly extends between germ cells located further adluminal (black arrowheads). (**B**) Tubules with germ cell neoplasia in situ show several basally located brown stained dots arranged between degenerated germ cells and remaining Sertoli cells (arrows)—an exact assignment to one of the two cell types can only be made speculatively. A similar reaction pattern can be seen in the center of the tubule (white arrowheads) where displaced Sertoli cells are located. (**C**) Seminoma cells are negative, only remaining tubule-like structures that still contain Sertoli cells display a linear but interrupted staining pattern along the basal edge (arrows). Negative controls do not display any immunopositive reaction (**inserts**). Scale bars = 50 μm

In case of Cx43, a linear staining pattern appeared in tubules with NSP, which was mainly visible above spermatogonia—thus in the area of the BTB (Fig. 8A, arrows). In some areas, the signal also appeared to extend between GCs (spermatocytes) localized further adluminal (Fig. 8A, black arrowheads). Based on this staining pattern, the signal could be assigned to SCs on the one hand, but also to the membranes of said GCs on the other hand. In GCNIS tubules, particularly centrally located cells, which are most likely SCs that were displaced adluminal, showed an immunopositive signal (Fig. 8B, white arrowheads). In addition, areas in close vicinity to basally located GCNIS cells were also stained (Fig. 8B, arrows), whereby the reaction could originate from these cells as well as from remaining SCs. Again, the cytoplasm of the GCNIS cells could not be assessed. The typical vesicular-looking seminoma cells were negative for Cx43, while in some areas of the section, remaining tubule-like structures were partly stained (Fig. 8C). Within these tubules, SCs were still present in addition to degenerate GCs. A linear but interrupted staining pattern appeared along the outer edge of these tubule-like structures (Fig. 8C, arrows), which—as already described for GCNIS—could be assigned to SCs and/or degenerated GCs. The respective negative controls did not display any staining (Fig. 8, inserts).

As the staining patterns of N-cadherin and Cx43 in human biopsies representative of NSP, GCNIS or seminoma are already known from former studies (see Discussion), our biopsy results can be used to better assess the results obtained from FS1 and TCam-2 cells.

**Fig. 9:**
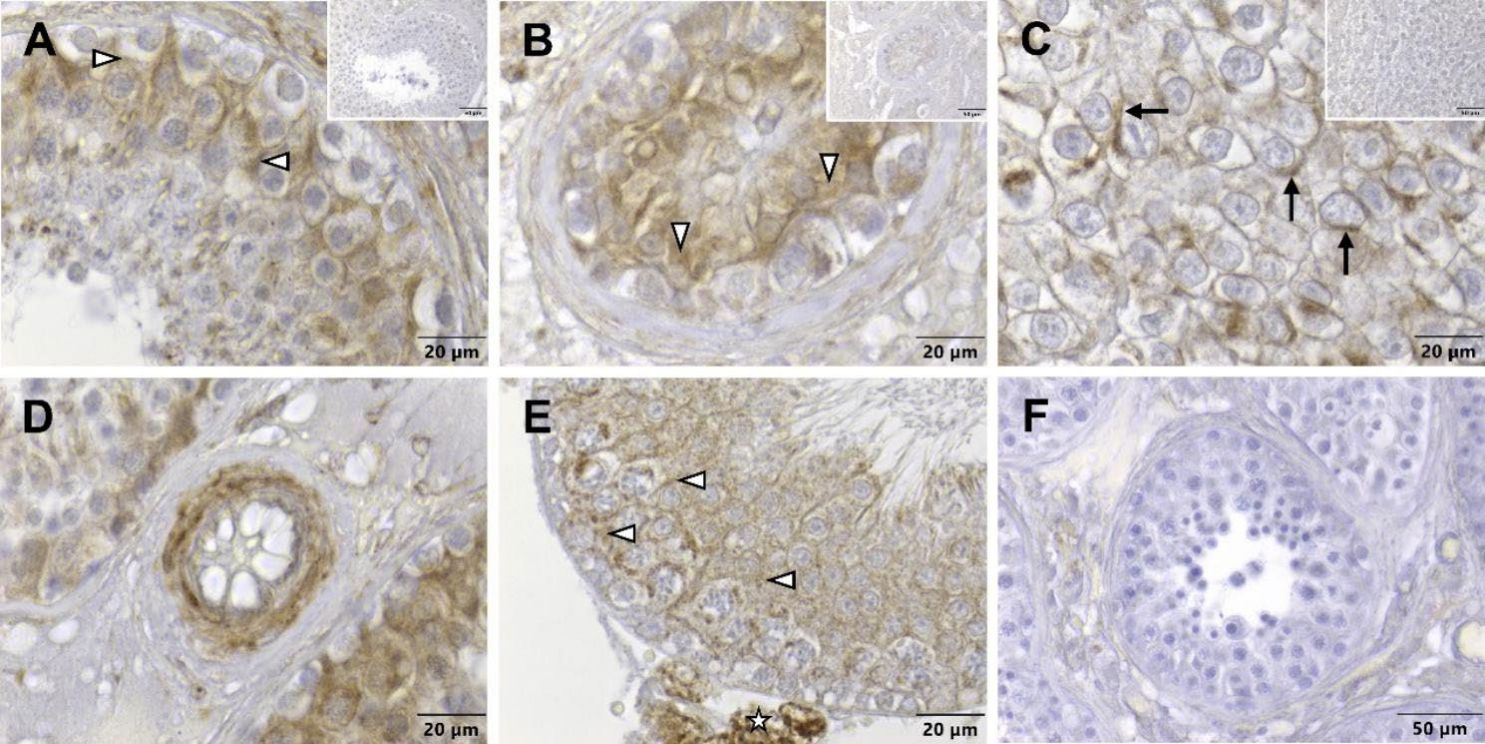
Immunostaining of connexin 45 in human testicular biopsy specimens. (**A**) In the tubule with normal spermatogenesis, the signal of the immunoreaction is clearly visible in the cytoplasm of Sertoli cells (white arrowheads) with the strongest signal intensity tending to be located in the basal half of the seminiferous epithelium. (**B**) In case of germ cell neoplasia in situ, the labelling is also attributed to the cytoplasm of Sertoli cells (white arrowheads), which are now located further adluminal. (**C**) Seminoma cells show a brown signal, which appears in spots rather than in lines, mainly at the boundaries to adjacent cells (arrows). Sertoli cells are not detectable anymore. (**D**) The wall of a blood vessel within a biopsy displaying normal spermatogenesis is clearly stained and can be interpreted as a positive control. (**E**) Within the seminiferous epithelium of an adult mouse with normal spermatogenesis, the cytoplasm of Sertoli cells is labelled (white arrowheads) as is the cytoplasm of Leydig cells (asterisk), which together additionally serves as a positive control. The sections treated with blocking peptide (**inserts**) as well as the representative negative control of a human normal spermatogenesis tubule (**F**) show no reaction. Scale bars = 20 μm (**A**–**E**), 50 μm (**F**; **inserts**)

Regarding Cx45, the cytoplasm of SCs in the seminiferous epithelium of a patient with NSP was found to be immunopositive (Fig. 9A, white arrowheads). The reaction mainly occurred in the basal part of the epithelium. In tubules with GCNIS, the labelling was mainly restricted to the cytoplasm of SCs that were displaced adluminal (Fig. 9B, white arrowheads). In case of seminoma, a spotty rather than linear signal was seen in the area of the cell boundaries of adjacent tumor cells, although it is difficult to say whether it is attributable to cytoplasmic remnants or to the cell membrane (Fig. 9C, arrows). On the one hand, a blood vessel’s wall of a patient with NSP served as a positive control (Fig. 9D), since it is known that vascular smooth muscle cells express Cx45 [47, 66]. On the other hand, brown staining of the cytoplasm of murine SCs [44] (Fig. 9E, white arrowheads) and LCs [46] (Fig. 9E, asterisks) can be considered as a positive control as well. Furthermore, the fact that a visible immune reaction could be prevented by the use of blocking peptide argues for the antibody’s specificity (Fig. 9, inserts). The representative negative control of a human normal spermatogenesis tubule (Fig. 9F) showed no immunopositive labelling.

## Discussion

Cell culture offers great opportunities for cancer research. Especially concerning GCTs, the restricted availability of fetal testicular material and the long period between initiation and progression limit investigations. Besides, there is no established animal model representing the aforementioned pathogenesis [51]. Recently, a coculture model consisting of the human SC line FS1 and the human seminoma-like cell line TCam-2 was developed [51, 58], which promises to be useful for elucidating the pathogenesis of seminoma. The aim of this study was to further characterize these two human cell lines focusing on junctional proteins and to compare them with human testicular material, as adhesion and communication between somatic SCs and tumor cells may influence cancer progression.

Early indications for the presence of adhesion structures in TCam-2 cells were provided by Eckert et al. who suspected a high amount of desmosomes due to the strong adherence to the tissue culture surface [55]. Further evidence was delivered by gene expression profiling, which showed that genes involved in junctional signaling were significantly overrepresented in TCam-2 cells [67]. Regarding the FS1 cell line, nothing is known about the formation of junctions yet, but it has already been emphasized that there is a need to examine cell interactions [50].

In the present study, N-cadherin was detected at both mRNA and protein level in FS1 and TCam-2 cells. Within FS1 cells, N-cadherin was prominently localized at the cell membranes and to a lesser extent in the cytoplasm. Similarly, in tubules with NSP, N-cadherin was present in the cytoplasm of SCs but was strongly evident at the cell boundaries between SCs and some GCs, as previously described by others [14, 15, 17, 18]. Thus, the expression of N-cadherin in FS1 cells resembles the expression of N-cadherin in SCs of the NSP tubule in the setting of methods we performed. In TCam-2-cells, a mainly membrane-associated but also a weak cytoplasmic staining could be observed for N-cadherin, thus bringing our findings into line with previous findings [16, 23]. Similarly, sections of seminoma showed the typical honeycomb-like expression—a well-known pattern which results from the expression of N-cadherin at the cell membranes of neighboring cells [14, 16, 17]—and thus can be used to confirm the results obtained from the TCam-2 cell line. However, the observed signal at the cell membranes of TCam-2 cells was relatively weak compared to FS1 cells and the expression value of N-cadherin was low in TCam-2 but high in FS1 cells. Other researchers claim that TCam-2 cells do not establish distinct AJs under basal conditions, even though some cadherin protein is detectable. They further suggest that external chemical or physical factors are required to stimulate de novo biosynthesis and incorporation of cadherins into the membrane by modulating cell metabolism [68, 69]. Nevertheless, these authors did not focus on N-cadherin, so different antibodies were used, and as an ultrastructural examination was not performed in the present study, the formation of AJs could not be evaluated. Whether the weak expression of N-cadherin localized in the cytoplasm of TCam-2 cells is also reflected by seminoma cells cannot be assessed, as the cytoplasm of these tumor cells contains much glycogen [52], which gets lost during immunochemical treatment on paraffin sections. However, since we also found a cytoplasmic expression of N-cadherin in FS1 cells and SCs, it can be assumed that seminoma cells also express N-cadherin in their cytoplasm, because cadherins—as is typical for proteins in general—are assembled in the endoplasmic reticulum after synthesis and then transported to the Golgi apparatus [70]. It is therefore likely that the cytoplasmic signal refers to N-cadherin that has not yet been integrated into the membrane. Alternatively, AJs could have been degraded by endocytosis, because it has already been shown that e.g. calcium depletion has an influence on the cytoskeleton of cultured cells resulting in retraction of the entire junctional complex [71, 72].

Some members of the cadherin family act as tumor suppressors while others promote cancer development; the latter applies to N-cadherin. Numerous studies have shown that upregulation of N-cadherin is significantly associated with lymph node metastases, histological grade, angiolymphatic invasion and clinical stage, suggesting that N-cadherin indicates an aggressive tumor and predicts poor survival [73]. An increase in N-cadherin is characteristic for epithelial-mesenchymal transition, which in turn contributes significantly to tumor progression [20, 21]. It is known that N-cadherin promotes motility and migration of tumor cells [74, 75], and after cells have entered the bloodstream, the adhesive properties of N-cadherin cause them to stick to the endothelium [22]. In seminoma [14, 16, 17] and in TCam-2 cells [16, 23] N-cadherin occurs, especially at the cell borders, a finding that we could confirm. Downregulation of N-cadherin in TCam-2 cells in vitro using blocking peptides or siRNA leads to a significant inhibition of proliferation, migration and invasion of TCam-2 cells and higher rates of apoptotic cells in caspase-3 staining [23]. Summed up, it is likely that the expression of N-cadherin in seminoma cells and in their representatives, TCam-2 cells, may be indicative for malignant potential, but further studies are necessary to confirm and define the exact role of N-cadherin in seminoma progression. Moreover, the presence of N-cadherin also promises diagnostic and prognostic benefits as different GCTs are known to show differences in cadherin expression [14, 16, 76]. Similarly, the expression pattern of N-cadherin in relation to other cadherins could provide information about the differentiation state of malignant GCTs [16], and furthermore, it may represent an important therapeutic target [77]. Overall, TCam-2 cells might be helpful to further analyze these hypotheses, as the expression pattern of N-cadherin in TCam-2 cells is very similar to that already known for seminoma, which was also obtained within the present study.

Communication via GJs consisting of connexins has a regulatory effect on cell proliferation and differentiation [24, 78], making them important for the maintenance of normal cell growth. At the same time, they constitute a family of tumor suppressor genes [79]. It was shown that Cx43 immunostaining is localized at the junctional complexes between SCs in tubules with NSP, but tubules infiltrated with GCNIS or seminoma display altered or no Cx43 expression [29, 33, 80]. At mRNA level, Cx43 is downregulated in SCs and GCs in seminiferous tubules containing GCNIS and completely disappears in seminoma cells, suggesting that the downregulation of Cx43 already takes place at mRNA level, starting with the infiltration of first GCNIS cells [29]. In the present study, FS1 cells showed positive results for Cx43 at both mRNA and protein level. A punctate to linear signal was visible at the cell membranes and dye-transfer provided functional coupling between neighboring FS1 cells in 79%. Thus, FS1 cells appear to communicate via functional Cx43-containing GJs, which corresponds to the situation in the normal adult human seminiferous tubule. Nevertheless, the origin of the FS1 cells must not be ignored. They derive from an adult (19 year old) patient with Frasier Syndrome, which is caused by a mutation in the Wilm’s tumor gene 1 (WT1), resulting in a decrease in WT1 + KTS isoforms due to a disruption of alternative splicing associated with decreased expression of the transcription factors SRY (sex-determining region on Y) and SOX9 (SRY box protein 9) in SCs, affecting their proper maturation [50]. A recent study demonstrated, using knockdown of WT1 and non-canonical Wnt signaling inhibitors in cultured bovine SCs, that WT1 negatively regulates the expression of Cx43 via the non-canonical pathway and thus is involved in SC differentiation [81]. In particular, the authors suggested the Wnt/Ca^2+^ pathway to be involved in regulating Cx43, which also effects cell adhesion. Thus, it is possible that the underlying mutation in the WT1 gene could determine the differentiation and adhesion state of FS1 cells and in particular the expression of Cx43. Nevertheless, the so far known marker profile of FS1 cells in its entirety corresponds to that of an adult SC [50, 51], with the +/-KTS isoform ratio being reversed and SOX9 expression being relatively low [50]. Furthermore, our results show clear parallels between the expression pattern of the pubertal marker Cx43 [28] in FS1 cells and adult SCs, as described above. It is important to consider that in humans, the WT1 mutation results in a reduction but not complete loss of the + KTS isoform and that WT1 also continues to be expressed [50]. Thus, whether or to what extent the expression of junctional proteins in FS1 cells, particularly Cx43, is determined by the mutation in the WT1 gene cannot be said at the present time, but the results of previous studies together with our results indicate an adult state of differentiation for FS1 cells.

When examining GCNIS, only at very few sites a residual reaction for Cx43 was still visible near the basement membrane, but an additional signal was conspicuous in the center of the tubule where the displaced SCs were located. Seminoma cells were found to be negative in the immunohistochemical examination, but again the cytoplasm could not be elucidated. Overall, the results for Cx43 obtained from human biopsies with GCNIS or seminoma are similar to those already described by others [29, 33] and therefore can be used to evaluate the results from TCam-2 cells. The staining of TCam-2 cells was very weak and rather localized in the cytoplasm. This is in accordance with the study of Ferranti et al. who detected Cx43 protein in TCam-2 cells under standard culture conditions in the perinuclear compartment, probably at the Golgi apparatus [69]. It was further speculated that seminoma may be characterized by an aberrant Cx43 localization in the Golgi apparatus and that this delocalization may participate in tumor progression [80]. Nevertheless, the performed dye-transfer experiments for TCam-2 cells revealed functional cell coupling in 56% of the studied cells. Thus, it might be assumed that Cx43 is present at least at low levels in the cytoplasm of TCam-2 cells and that there is functional coupling via GJs. The multiple immunoreactive bands between about 39 and 45 kDa, which occurred during Western blot analysis, probably indicate differently phosphorylated isoforms of Cx43 [29, 82]. Although we have not performed further investigations regarding the phosphorylation status, it is believed that Cx43 becomes phosphorylated when integrated into the membrane [82], which argues for the formation of GJs consisting of Cx43 at least in FS1 cells. Based on these results, it stands to reason that another connexin is involved in the formation of GJs in TCam-2 cells.

Against this background, also Cx45 was investigated in the present study. The expression could be detected at mRNA and protein level and could be localized in the cytoplasm of FS1 and TCam-2 cells. Thus, for FS1 cells the expression pattern of Cx45 matches the situation in situ, as SCs in tubules with NSP and with GCNIS similarly exhibited a cytoplasmic immunoreaction. There are no studies on the presence of Cx45 in the human testis to our knowledge up to now, but some human databases provide evidence for the presence of Cx45 in the human testis at the gene level [83, 84]. At present, it is not possible to say what function Cx45 assumes in SCs or FS1 cells. The results of the present study indicate that Cx43 rather than Cx45 seems to be involved in the formation of GJs. Seminoma cells showed a labelling that could not be assigned reliably to either the membrane or the cytoplasm by means of immunohistochemical examination alone. Even though Cx45 could not be found at the cell membranes of TCam-2 cells either, it is quite conceivable that Cx45-GJs are yet present—as it is known that Cx45-containing GJ plaques are very small [85]—especially since dye-transfer experiments revealed that some TCam-2 cells are actually coupled to each other. Furthermore, it is thinkable that Cx45 located in the cytoplasm could also have an influence on e.g. cell growth, but the current knowledge about the occurrence and function of Cx45 in tumor cells is rather limited. Similar to the present study’s outcome, a cytoplasmic rather than a membrane localization of Cx45 was found in tumor cells of malignant cardiac sarcoma [86] and oral squamous cell carcinoma [87]. So far, however, no association between Cx45 and the pathogenesis or behavior of a tumor could be shown.

Thus, although gap junction intercellular communication (GJIC) is not completely absent in TCam-2 cells, the membrane connexin expression appears to be at a low level. The loss of intact intercellular communication is closely linked to dedifferentiation, proliferation and invasion processes, and subsequently to tumor growth [37, 38, 88]. In case of GCNIS, it is not yet clear whether the disturbed intercellular communication is a consequence of proliferation or whether a common aetiopathological event is the trigger for both [33]. Since we only studied the connection between cells themselves and not the relationship between FS1 and TCam-2 cells yet, it can only been assumed that TCam-2 cells would escape the influence of FS1 cells in coculture. Especially GJ-protein Cx43 becomes further interesting for cancer therapy because it can act as a tumor suppressor [39]. Transfection experiments with Cx43 promise success, since both in vitro and in vivo proliferation, invasion and metastasis of tumor cells get suppressed, whereby GJIC is partially restored, which however does not seem to be necessary for the suppressive effect [80, 89–91]. An investigation of FS1 and TCam-2 cells to this end could provide a new therapeutic approach for GCTs.

Finally, considering junctions in the testis, the functionality of the BTB is always of interest. Cx43 is known to have a regulatory effect on BTB assembly and dynamics. Studies in this field showed that deletion of testicular Cx43 or blocking of GJs leads to an altered assembly of TJs and AJs, but the integrity of the BTB and its functionality are not affected [92–95]. Thus, a study of Carette et al. on mice with knockout of Cx43 in SCs only revealed increased synthesis of AJs and TJs, the former composed of N-cadherin among others, when Cx43 is absent and communication via GJs is disrupted [92]. Although these studies focus on SCs, one can speculate that the sparse expression of Cx43 and the membrane-associated expression of N-cadherin in seminoma and TCam-2 cells are correlated, as a regulatory influence of Cx43 is possibly decreased. Furthermore, our experiments revealed membrane-bound expression of both Cx43 and N-cadherin in FS1 cells, which provides first evidence for a barrier function. It is already known that BTB integrity becomes impaired during infiltration with tumor cells [96, 97]. In this context, Fink et al. detected adhesive complexes between neighboring SCs in GCNIS-tubules by electron microscopy, but the BTB was passable for lanthanum tracer [51]. Overall, the investigation of junctions within FS1 cells provides the basis for further research on the association between BTB integrity and the pathogenesis of seminoma.

## Conclusion

It becomes obvious that many questions about human GCTs are still unanswered, spanning from the initiation of neoplastic transformation to the presence of a manifest metastatic tumor. A broad understanding of the mechanisms behind pathogenesis is fundamental for diagnostics, prognostics and therapy and is therefore paramount.

The results of this study show that Cx43, Cx45 and N-cadherin are generally present in both FS1 and TCam-2 cells at mRNA and/or protein level—in different amounts and localizations—and thus provide further approaches for investigating adhesion and communication in the context of testicular cancer development. Since both connexins and cadherins in are generally thought to have an influence on cancer progression, they represent important candidates for research in this field. Cell culture offers the advantage that no human tissue or no animal model is required and enables a wide range of investigations. As further evidence on the representativeness and suitability of FS1 and TCam-2 cells is provided by the present study, these human cell lines represent promising research tools for the future. We examined the selected junctional proteins on a primarily qualitative level to create a well-founded impression regarding general occurrence and localization. For the future, it would be interesting to analyze and compare the quantitative occurrence of these proteins and possible changes in their expression under various cancer-related conditions. Likewise, further studies are needed to determine the influence of cellular adhesion and communication behavior on seminoma progression and the underlying mechanisms. Thus, overall our data describe initial starting points to elucidate cell contacts and reciprocal relationship between somatic SCs and seminoma cells during subsequent coculture experiments.

## Electronic supplementary material

Below is the link to the electronic supplementary material.

Supplementary Material 1

Supplementary Material 2

Supplementary Material 3

## Data Availability

All data generated or analyzed during this study are included in this published article and its supplementary information files. Full primary microarray data are available at NCBI’s Gene Expression Omnibus, GEO Series accession number GSE169557, https://www.ncbi.nlm.nih.gov/geo/query/acc.cgi?acc=GSE169557 [51].

## Abbreviations

**AJ(s)**: Adherens junction(s)
**bp**: Base pair(s)
**BSA**: Bovine serum albumine
**BTB**: Blood-testis barrier
**cDNA**: Complementary DNA
**Cx**: Connexin
**DAPI**: 4′,6-diamidino-2-phenylindole
**DMEM**: Dolbecco’s Modified Eagles’s Medium
**DNA**: Deoxyribonucleic acid
**FCS**: Fetal calf serum
**GC(s)**: Germ cell(s)
**GCT(s)**: Germ cell tumor(s)
**GCNIS**: Germ cell neoplasia in situ
**GFP**: Green fluorescent protein
**GJ(s)**: Gap junction(s)
**GJIC**: Gap junction intercellular communication
**ICC**: Immunocytochemistry
**IF**: Immunofluorescence
**IHC**: Immunohistochemistry
**kDa**: Kilodalton
**LY**: Lucifer Yellow lithium salt
**mRNA**: Messenger RNA
**NSP**: Normal spermatogenesis
**PAGE**: Polyacrylamide gel electrophoresis
**PBS**: Phosphate-buffered saline
**RNA**: Ribonucleic acid
**RT**: Room temperature
**(RT-)PCR**: (Reverse transcription) polymerase chain reaction
**SC(s)**: Sertoli cell(s)
**SDS**: Sodium dodecyl sulfate
**SOX9**: SRY box protein 9
**SRY**: Sex-determining region on Y
**TBS(-T)**: Tris-buffered saline (with Tween)
**TJ(s)**: Tight junction(s)
**WB**: Western blot
**WT1**: Wilm’s tumor gene 1
