# Supplementary material for: Analysis of connexin 43, connexin 45 and N-cadherin in the human sertoli cell line FS1 and the human seminoma-like cell line TCam-2 in comparison with human testicular biopsies
Source: BMC Cancer. 2023 Mar 10;23:232. doi: 10.1186/s12885-023-10696-7 (PMC10007848; doi:10.1186/s12885-023-10696-7)
Supplement: Supplementary file 1 — Supplementary Material 1 [file 12885_2023_10696_MOESM1_ESM.docx]

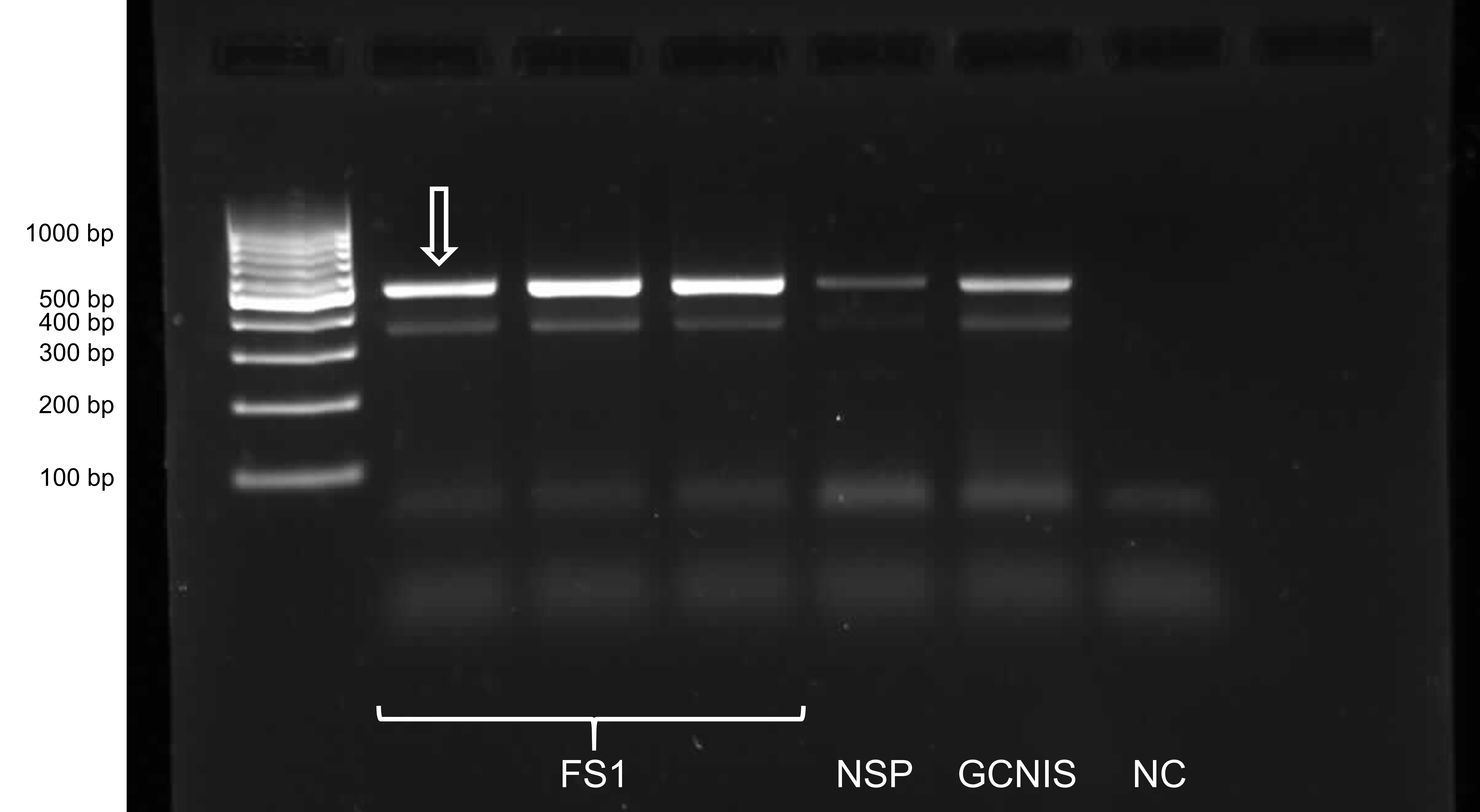


**Supplementary Fig. 1** Full-length uncropped gel for Fig. 1: Qualitative PCR analysis of N-cadherin in FS1 cells. Results for different samples of FS1 cells and human testicular biopsy specimens of patients with normal spermatogenesis (NSP) or germ cell neoplasia in situ (GCNIS) are shown. The PCR product representing N-cadherin can be observed as a band at 454 bp in all samples investigated. Other bands can be considered nonspecific. The band that is depicted in Fig. 1 of the main article is marked with an arrow. The negative control (NC) does not show any specific band.


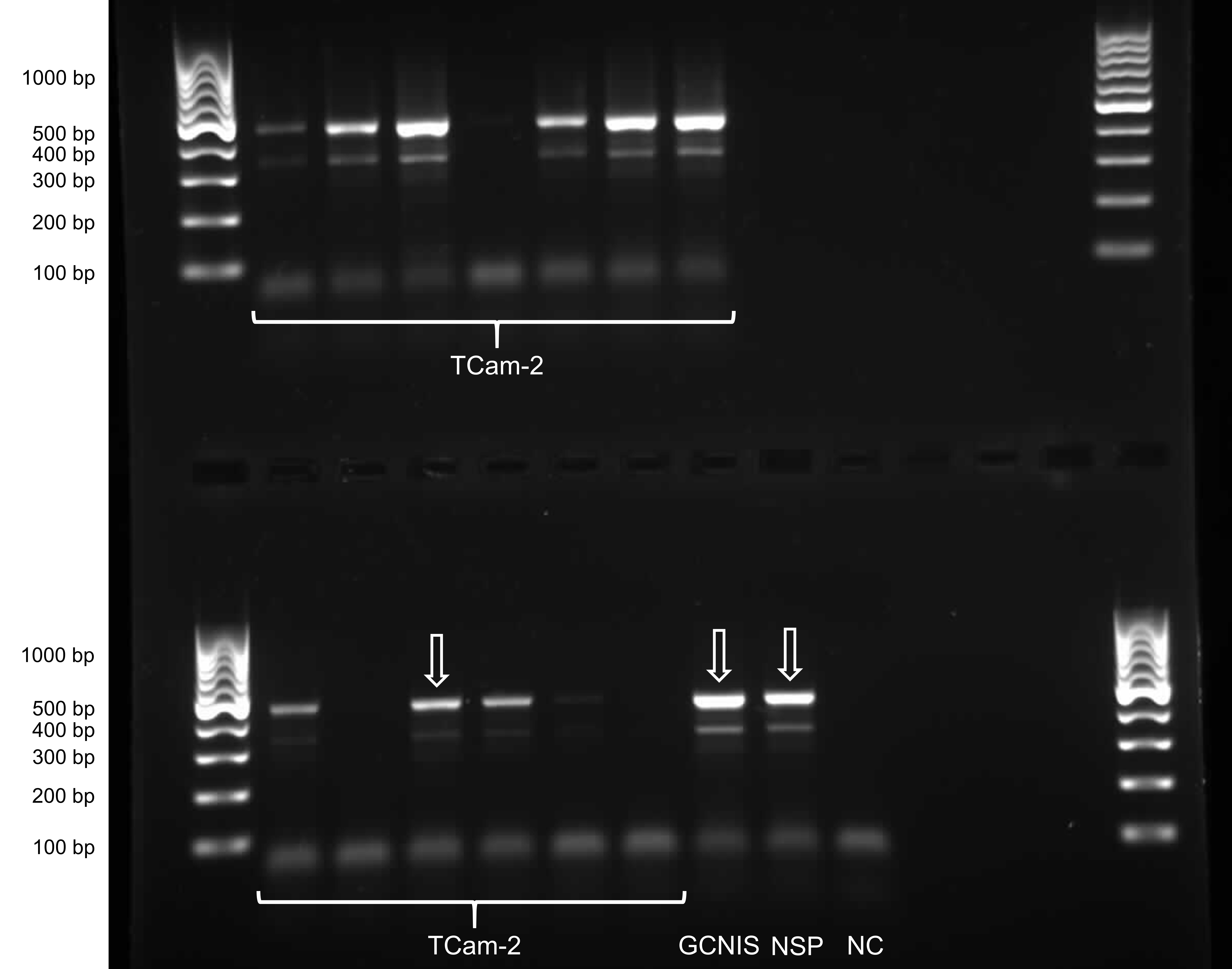


**Supplementary Fig. 2** Full-length uncropped gel for Fig. 1: Qualitative PCR analysis of N-cadherin in TCam-2 cells. Results for different samples of TCam-2 cells and human testicular biopsy specimens of patients with normal spermatogenesis (NSP) or germ cell neoplasia in situ (GCNIS) are shown. The PCR products representing N-cadherin can be observed as a band at 454 bp. Other bands can be considered nonspecific. Although not all but still most TCam-2 samples exhibit a respective PCR product at 454 bp, as it is also the case for GCNIS and NSP samples. The bands that are depicted in Fig. 1 of the main article are marked with arrows. The negative control (NC) does not show any specific band.


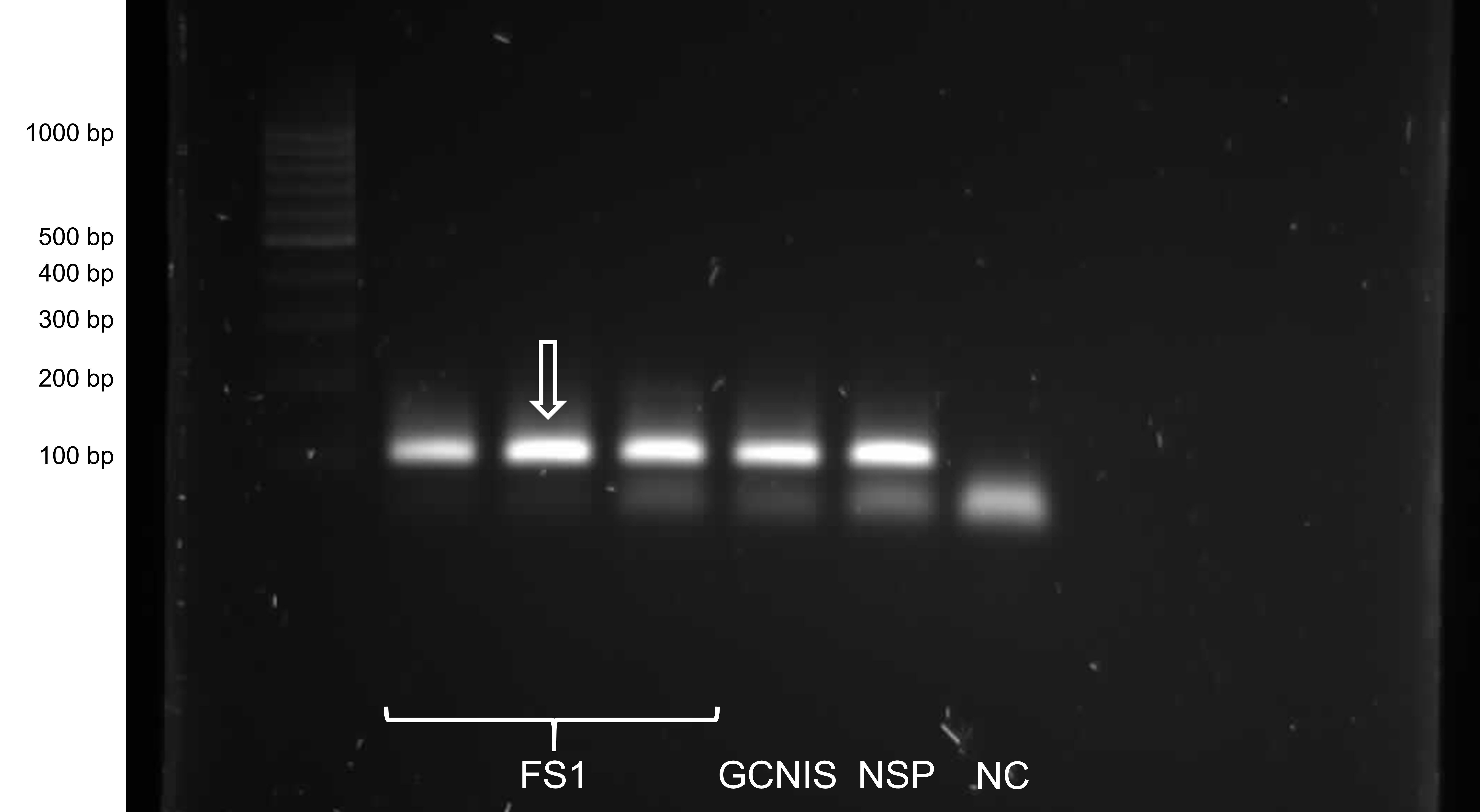


**Supplementary Fig. 3** Full-length uncropped gel for Fig. 1: Qualitative PCR analysis of connexin 43 in FS1 cells. Results for different samples of FS1 cells and human testicular biopsy specimens of patients with normal spermatogenesis (NSP) or germ cell neoplasia in situ (GCNIS) are shown. The PCR product representing connexin 43 can be observed as a band at 138 bp in all samples investigated. The band that is depicted in Fig. 1 of the main article is marked with an arrow. The negative control (NC) does not show any specific band.


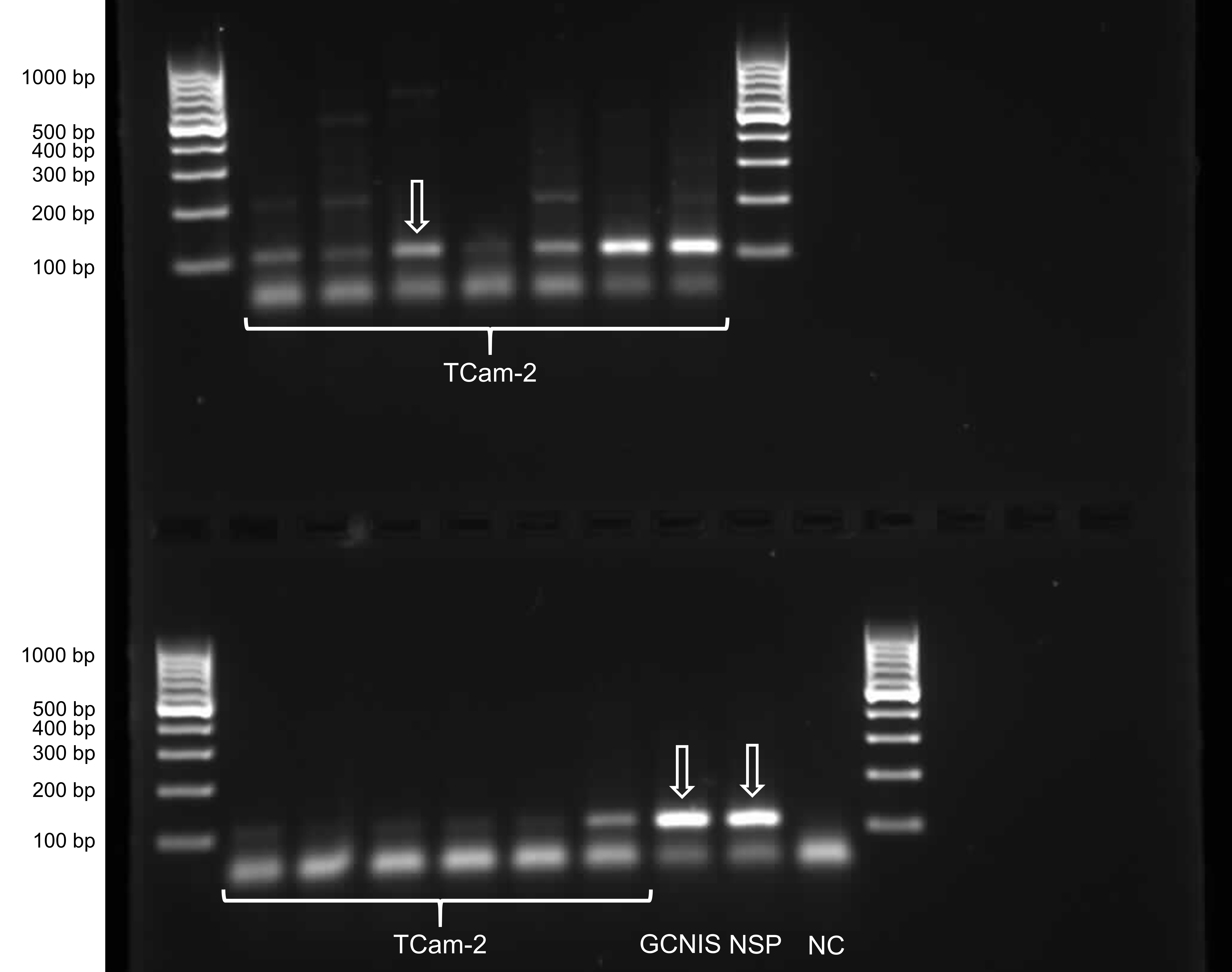


**Supplementary Fig. 4** Full-length uncropped gel for Fig. 1: Qualitative PCR analysis of connexin 43 in TCam-2 cells. Results for different samples of TCam-2 cells and human testicular biopsy specimens of patients with normal spermatogenesis (NSP) or germ cell neoplasia in situ (GCNIS) are shown. The PCR product representing connexin 43 can be observed as a band at 138 bp. Other bands can be considered nonspecific. Although not all but still most TCam-2 samples exhibit a respective PCR product at 138 bp, as it is also the case for GCNIS and NSP samples. The bands that are depicted in Fig. 1 of the main article are marked with arrows. The negative control (NC) does not show any specific band.


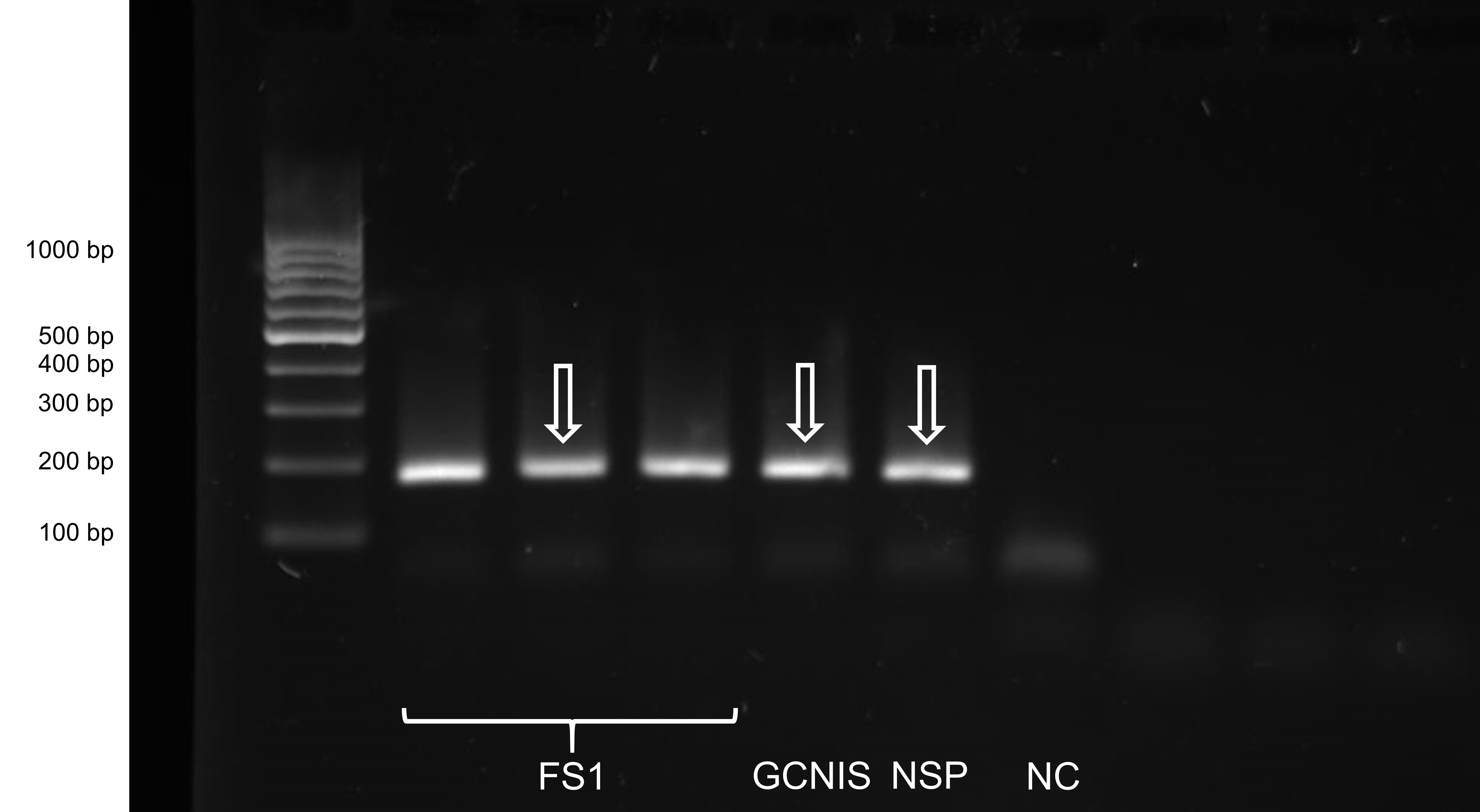


**Supplementary Fig. 5** Full-length uncropped gel for Fig. 1: Qualitative PCR analysis of connexin 45 in FS1 cells. Results for different samples of FS1 cells and human testicular biopsy specimens of patients with normal spermatogenesis (NSP) or germ cell neoplasia in situ (GCNIS) are shown. The PCR product representing connexin 45 can be observed as a band at 174 bp in all samples investigated. The bands that are depicted in Fig. 1 of the main article are marked with arrows. The negative control (NC) does not show any specific band.


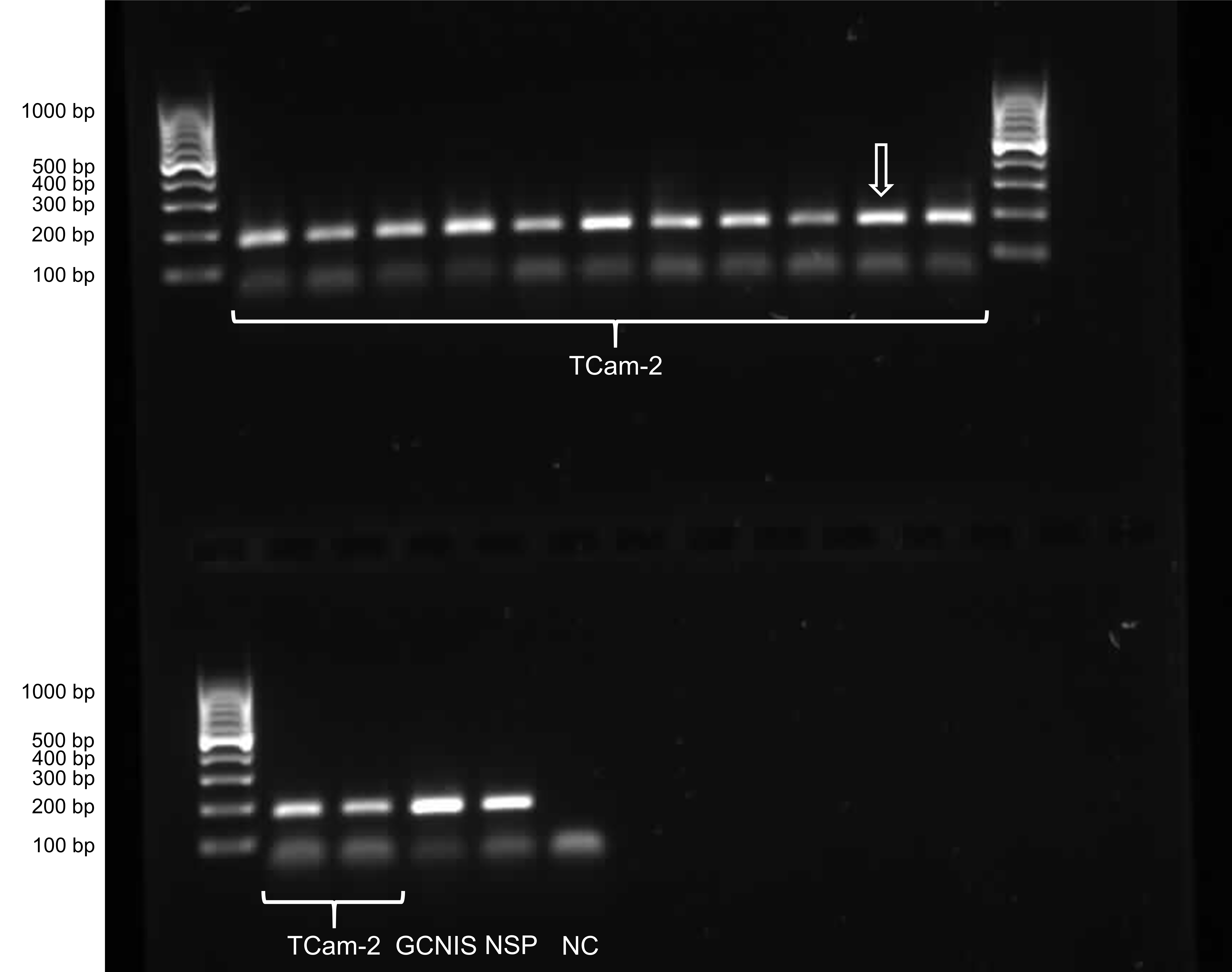


**Supplementary Fig. 6** Full-length uncropped gel for Fig. 1: Qualitative PCR analysis of connexin 45 in TCam-2 cells. Results for different samples of TCam-2 cells and human testicular biopsy specimens of patients with normal spermatogenesis (NSP) or germ cell neoplasia in situ (GCNIS) are shown. The PCR product representing connexin 45 can be observed as a band at 174 bp in all samples investigated. The band that is depicted in Fig. 1 of the main article is marked with an arrow. The negative control (NC) does not show any specific band.


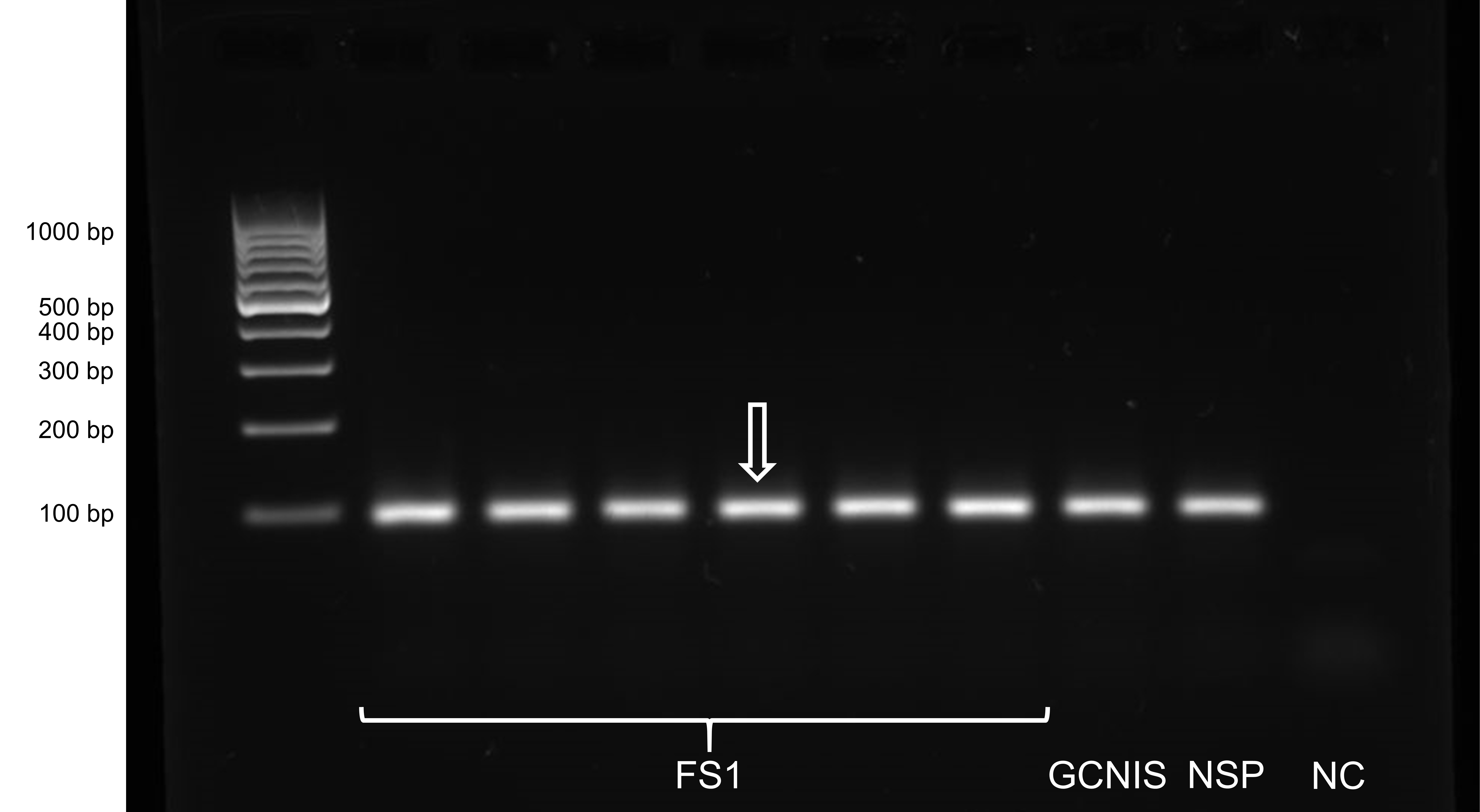


**Supplementary Fig. 7** Full-length uncropped gel for Fig. 1: Qualitative PCR analysis of beta-actin in FS1 cells. Results for different samples of FS1 cells and human testicular biopsy specimens of patients with normal spermatogenesis (NSP) or germ cell neoplasia in situ (GCNIS) are shown. The PCR products representing beta-actin can be observed as a band at 90 bp in all samples inbestigated. The band that is depicted in Fig. 1 of the main article is marked with an arrow. The negative control (NC) does not show any specific band.


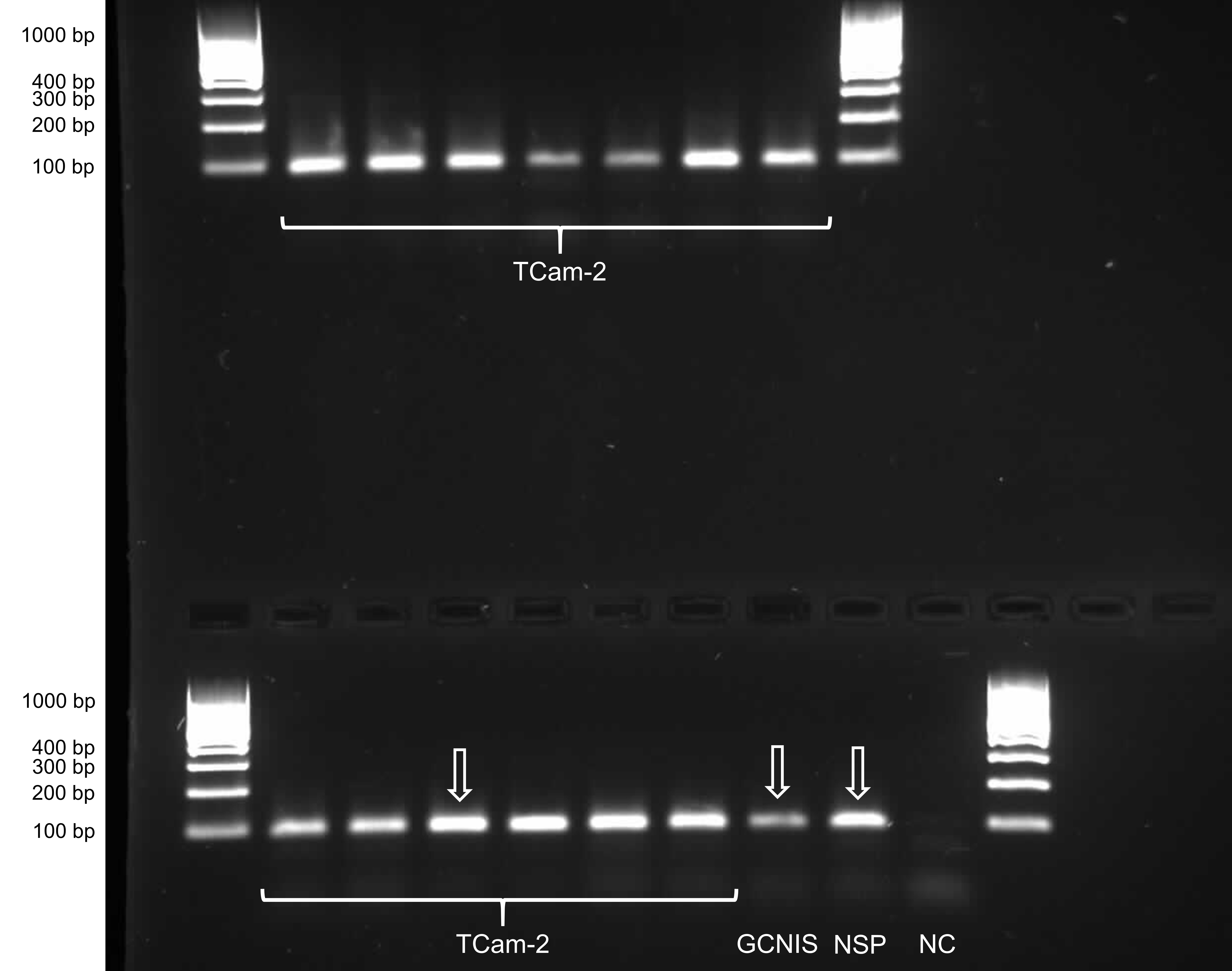


**Supplementary Fig. 8** Full-length uncropped gel for Fig. 1: Qualitative PCR analysis of beta-actin in TCam-2 cells. Results for different samples of TCam-2 cells and human testicular biopsy specimens of patients with normal spermatogenesis (NSP) or germ cell neoplasia in situ (GCNIS) are shown. The PCR products representing beta-actin can be observed as a band at 90 bp in all samples investigated. The bands that are depicted in Fig. 1 of the main article are marked with arrows. The negative control (NC) does not show any specific band.
