# Supplementary material for: Analysis of connexin 43, connexin 45 and N-cadherin in the human sertoli cell line FS1 and the human seminoma-like cell line TCam-2 in comparison with human testicular biopsies
Source: BMC Cancer. 2023 Mar 10;23:232. doi: 10.1186/s12885-023-10696-7 (PMC10007848; doi:10.1186/s12885-023-10696-7)
Supplement: Supplementary file 2 — Supplementary Material 2 [file 12885_2023_10696_MOESM2_ESM.docx]

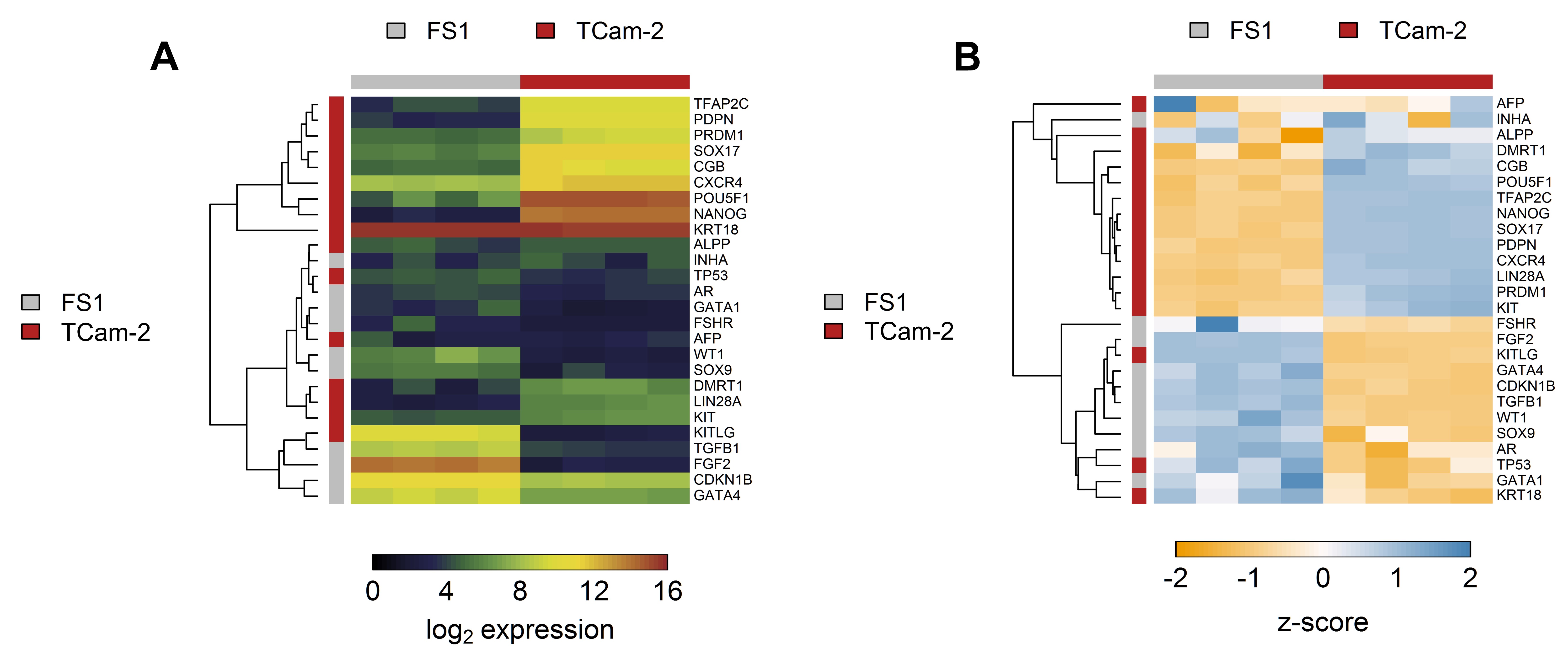
**Supplementary Fig. 9** Expression of certain known marker genes in FS1 and TCam-2 cells. The heatmaps show results for the microarray analysis of monocultured FS1 and TCam-2 cells. Columns represent biological replicates of either FS1 or TCam-2 cells, and rows represent a selection of expressed marker genes typical for adult Sertoli/FS1 cells and seminoma/TCam-2 cells, respectively. Heatmap (**A**) shows expression values and heatmap (**B**) shows z-scores.
