# Supplementary material for: Analysis of connexin 43, connexin 45 and N-cadherin in the human sertoli cell line FS1 and the human seminoma-like cell line TCam-2 in comparison with human testicular biopsies
Source: BMC Cancer. 2023 Mar 10;23:232. doi: 10.1186/s12885-023-10696-7 (PMC10007848; doi:10.1186/s12885-023-10696-7)
Supplement: Supplementary file 3 — Supplementary Material 3 [file 12885_2023_10696_MOESM3_ESM.docx]

**Supplementary Fig. 10** Full-length Western blot for Fig. 3: Qualitative analysis of N-cadherin in FS1 and TCam-2 cells. Results for different FS1 and TCam-2 samples are presented. Every picture shows the same membrane. In images (**A.1**) and (**A.2**), the respective marker is visible after white light exposure. Images (**B.1**), (**C.1**), (**D.1**) and (**E.1**) show the results for N-cadherin, after exposure for 27 s (**B.1**, **C.1**) and 1 min and 30 s (**D.1**, **E.1**), respectively, whereby images (**C.1**) and (**E.1**) represent inverted versions for better contrast. Images (**B.2**), (**C.2**), (**D.2**) and (**E.2**) show the corresponding results for the loading control beta-actin, after the membrane was treated with stripping buffer, at an exposure of 29 s (**B.2**, **C.2**) and 1 min and 30 s (**D.2**, **E.2**), respectively. Again, images (**C.2**) and (**E.2**) are inverted versions. The immunoreactive band representing N-cadherin can be observed at 140 kDa for FS1 and TCam-2 samples (**B.1**, **C.1**, **D.1**, **E.1**). The typical band for beta-actin is located at 43 kDa and is also present for FS1 and TCam-2 samples (**B.2**, **C.2**, **D.2**, **E.2**). The bands that are depicted in Fig. 3 of the main article are marked with arrows.


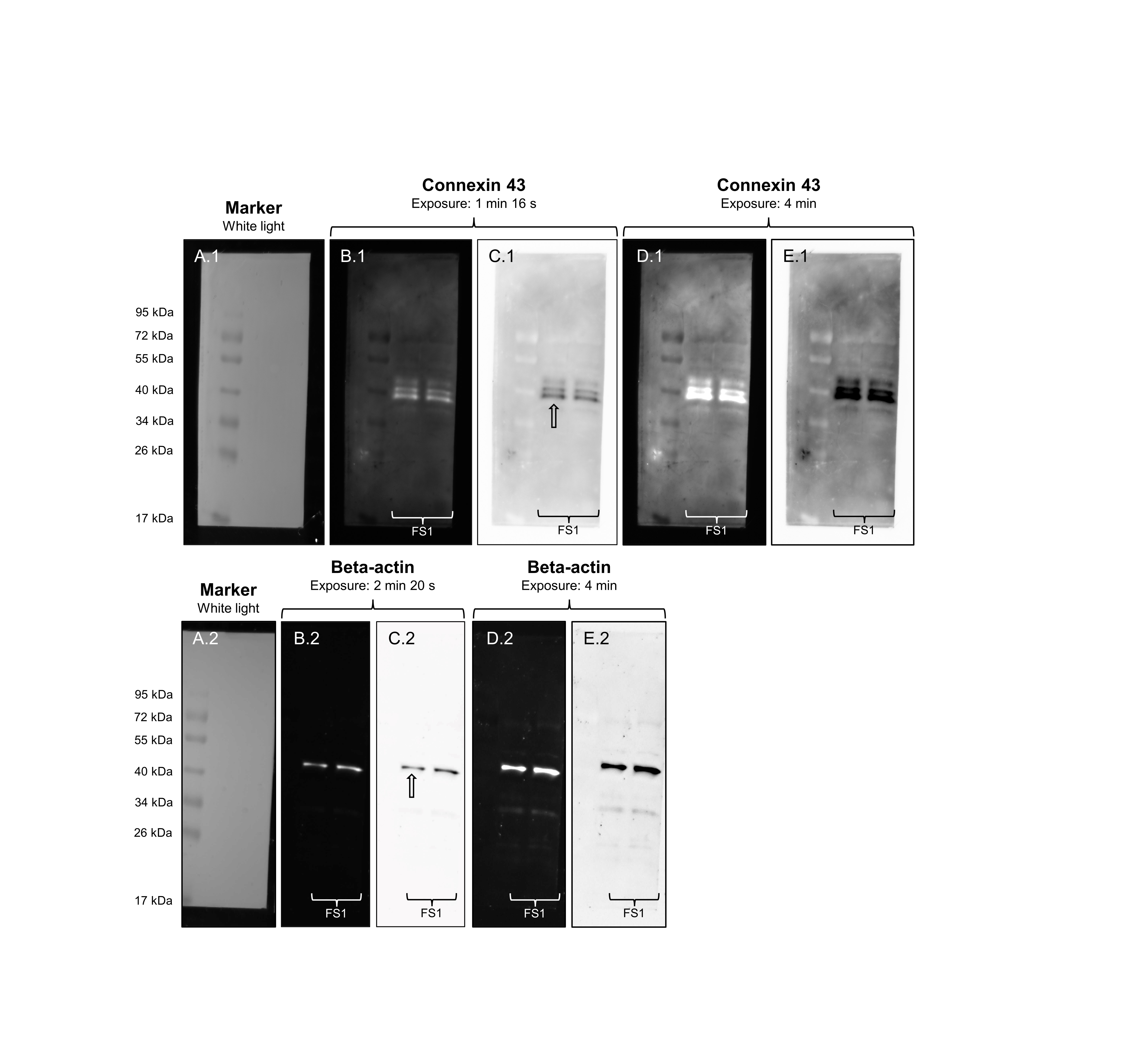


**Supplementary Fig. 11** Full-length Western blot for Fig. 3: Qualitative analysis of connexin 43 in FS1 cells. Results for two different FS1 samples are presented. Every picture shows the same membrane. In images (**A.1**) and (**A.2**), the respective marker is visible after white light exposure. Images (**B.1**), (**C.1**), (**D.1**) and (**E.1**) show the results for connexin 43, after exposure for 1 min and 16 s (**B.1**, **C.1**) and 4 min (**D.1**, **E.1**), respectively, whereby images (**C.1**) and (**E.1**) represent inverted versions for better contrast. Images (**B.2**), (**C.2**), (**D.2**) and (**E.2**) show the corresponding results for the loading control beta-actin, after the membrane was treated with stripping buffer, at an exposure of 2 min 20 s (**B.2**, **C.2**) and 4 min (**D.2**, **E.2**), respectively. Again, images (**C.2**) and (**E.2**) are inverted versions. The immunoreactive bands representing connexin 43 can be observed at 39 kDa, 41 kDa and 43/44 kDa for FS1 samples, probably representing different phosphorylated isoforms (**B.1**, **C.1**, **D.1**, **E.1**). The typical band for beta-actin is located at 43 kDa and is also present for FS1 samples (**B.2**, **C.2**, **D.2**, **E.2**). The bands that are depicted in Fig. 3 of the main article are marked with arrows.


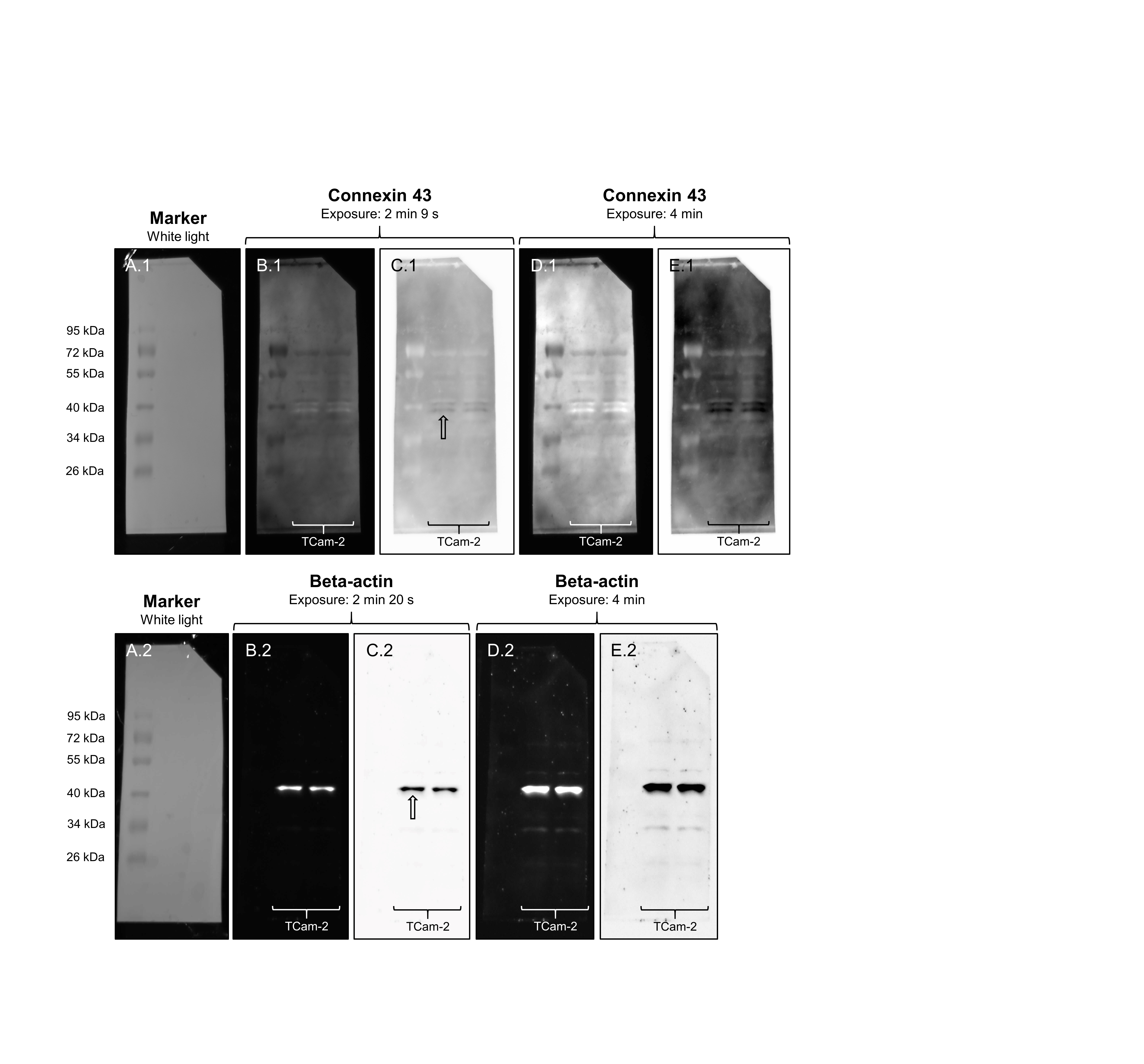


**Supplementary Fig. 12** Full-length Western blot for Fig. 3: Qualitative analysis of connexin 43 in TCam-2 cells. Results for two different TCam-2 samples are presented. Every picture shows the same membrane. In images (**A.1**) and (**A.2**), the respective marker is visible after white light exposure. Images (**B.1**), (**C.1**), (**D.1**) and (**E.1**) show the results for connexin 43, after exposure for 2 min and 9 s (**B.1**, **C.1**) and 4 min (**D.1**, **E.1**), respectively, whereby images (**C.1**) and (**E.1**) represent inverted versions for better contrast. Images (**B.2**), (**C.2**), (**D.2**) and (**E.2**) show the corresponding results for the loading control beta-actin, after the membrane was treated with stripping buffer, at an exposure of 2 min and 20 s (**B.2**, **C.2**) and 4 min (**D.2**, **E.2**), respectively. Again, images (**C.2**) and (**E.2**) are inverted versions. The immunoreactive bands representing connexin 43 can be observed at 39 kDa and 41 kDa for TCam-2 samples (**B.1**, **C.1**, **D.1**, **E.1**). The typical band for beta-actin is located at 43 kDa and is also present for TCam-2 samples (**B.2**, **C.2**, **D.2**, **E.2**). The bands that are depicted in Fig. 3 of the main article are marked with arrows.


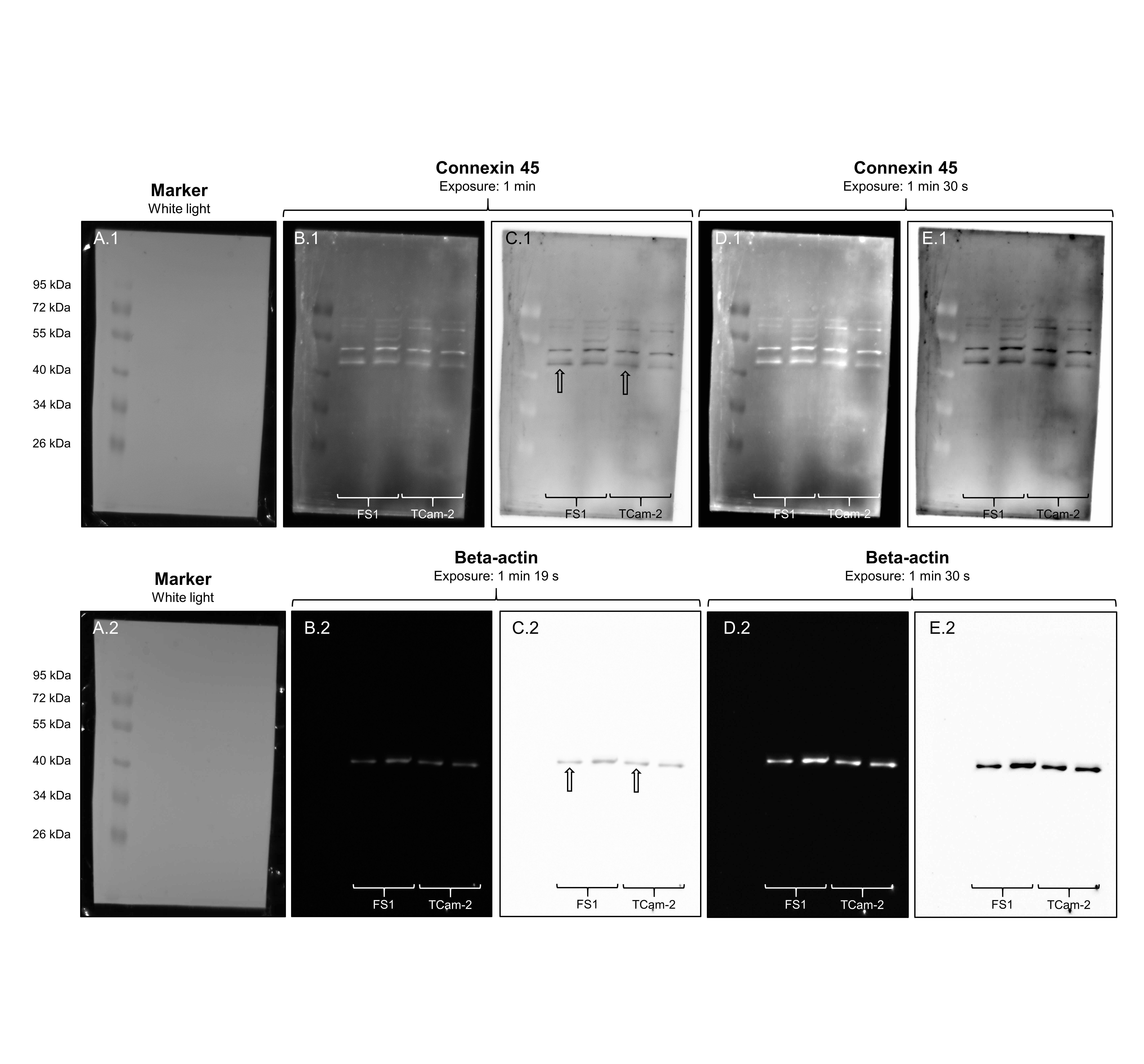


**Supplementary Fig. 13** Full-length Western blot for Fig. 3: Qualitative analysis of connexin 45 in FS1 and TCam-2 cells. Results for different FS1 and TCam-2 samples are presented. Every picture shows the same membrane. In images (**A.1**) and (**A.2**), the respective marker is visible after white light exposure. Images (**B.1**), (**C.1**), (**D.1**) and (**E.1**) show the results for connexin 45, after exposure for 1 min (**B.1**, **C.1**) and 1 min and 30 s (**D.1**, **E.1**), respectively, whereby images (**C.1**) and (**E.1**) represent inverted versions for better contrast. Images (**B.2**), (**C.2**), (**D.2**) and (**E.2**) show the corresponding results for the loading control beta-actin, after the membrane was treated with stripping buffer, at an exposure of 1 min and 19 s (**B.2**, **C.2**) and 1 min and 30 s (**D.2**, **E.2**), respectively. Again, images (**C.2**) and (**E.2**) are inverted versions. The immunoreactive bands representing connexin 45 can be observed at 45 kDa for FS1 and TCam-2 samples (**B.1**, **C.1**, **D.1**, **E.1**). The typical band for beta-actin is located at 43 kDa and is also present for FS1 and TCam-2 samples (**B.2**, **C.2**, **D.2**, **E.2**). Additional bands can be considered nonspecific. The bands that are depicted in Fig. 3 of the main article are marked with arrows.


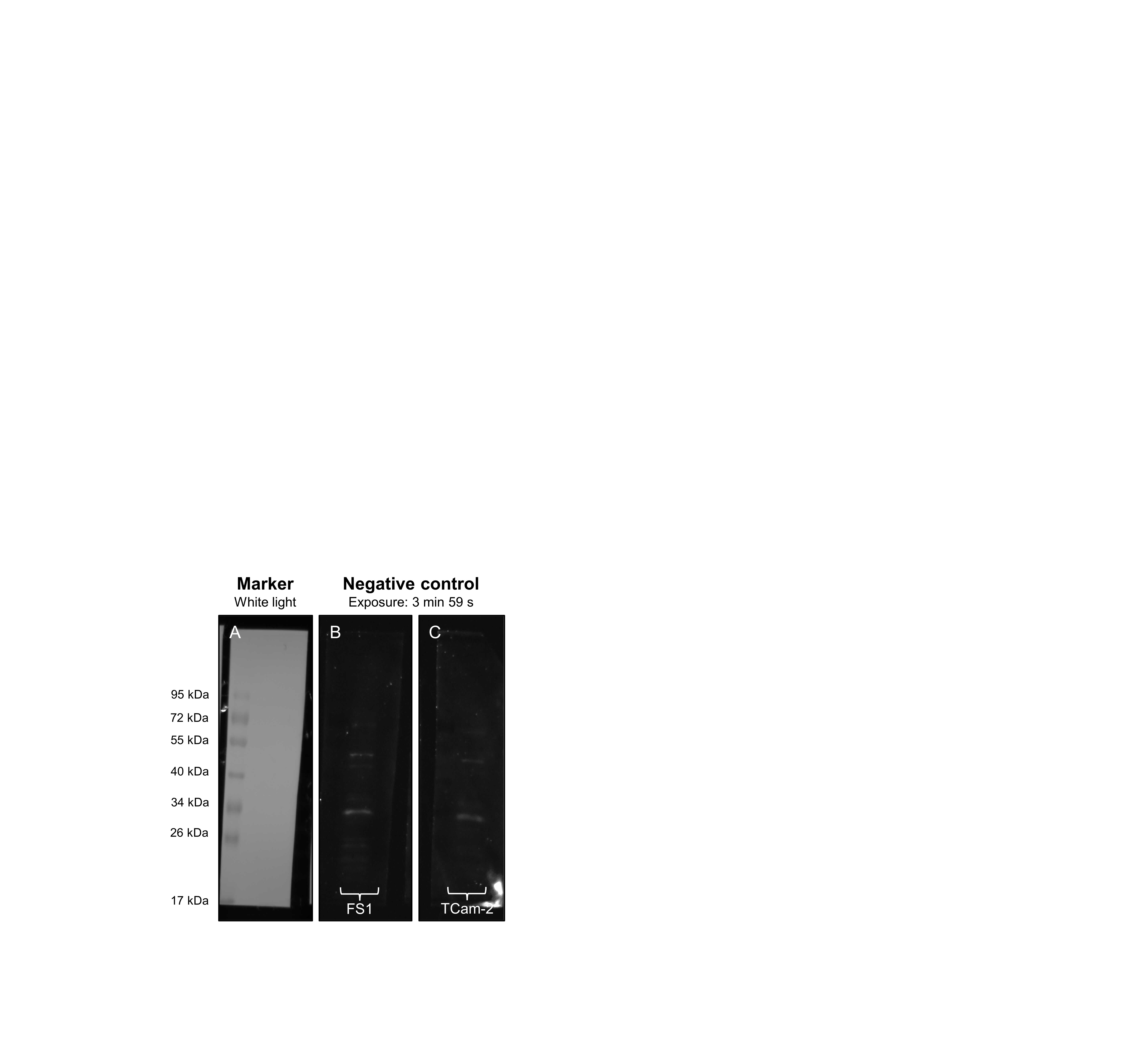


**Supplementary Fig. 14** Western blot representing negative controls (anti-mouse) for FS1 and TCam-2 cells. Only anti-mouse secondary antibody has been used but no primary antibody. Image (**A**) shows the marker after white light exposure and images (**B**) and (**C**) show the negative controls for FS1 and TCam-2 cells, respectively, after exposure for 3 min and 59 s. (**B**, **C**) Only faint bands can be observed, which can be considered nonspecific.


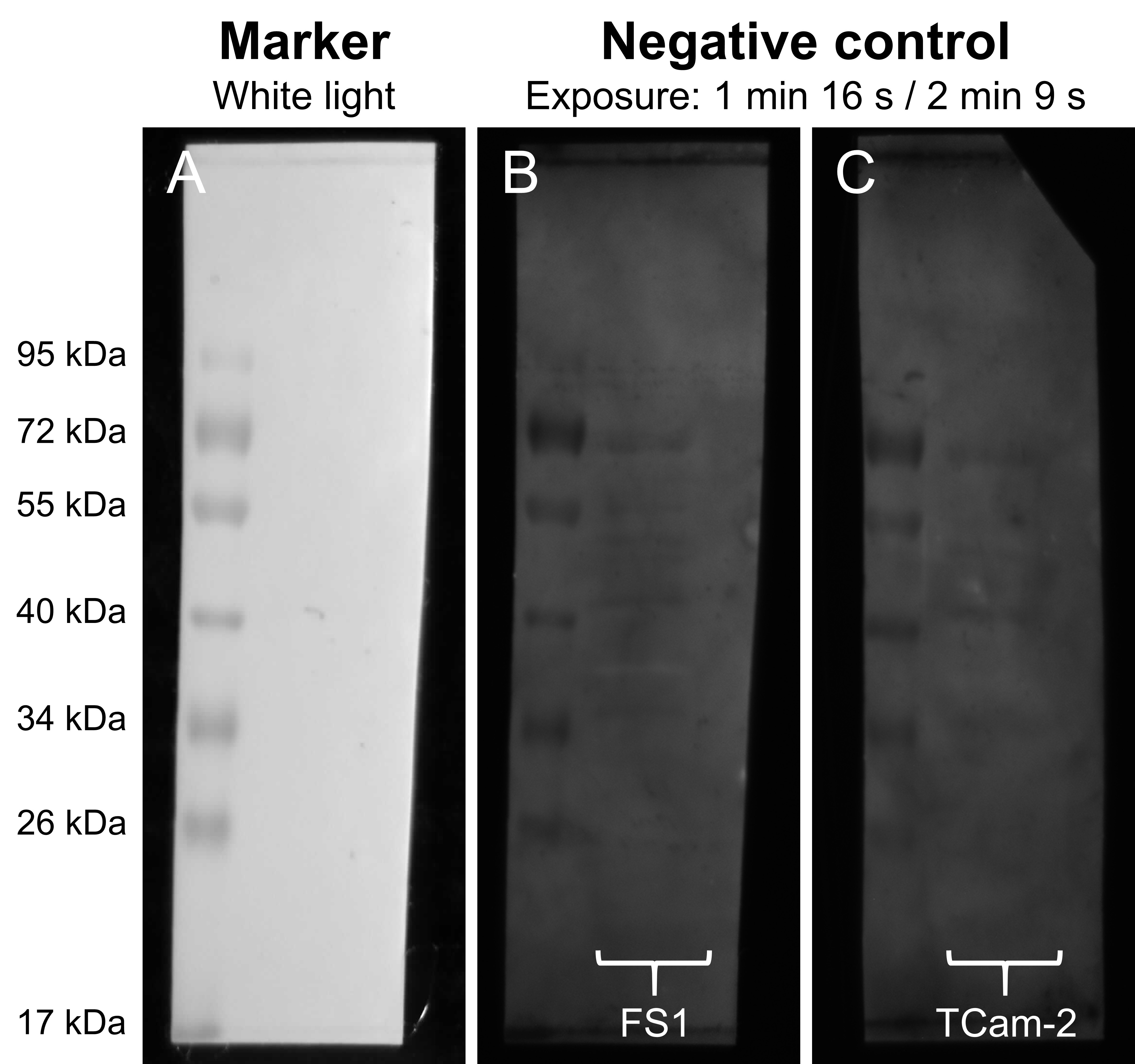


**Supplementary Fig. 15** Western blot representing negative controls (anti-rabbit) for FS1 and TCam-2 cells. Only anti-rabbit secondary antibody has been used but no primary antibody. Image (**A**) shows the marker after white light exposure and images (**B**) and (**C**) show the negative controls for FS1 (exposure: 1 min 16 s) and TCam-2 cells (exposure: 2 min 9 s), respectively. (**B, C**) No immunoreactive bands can be observed.
